# Controlling the Formation of Two Concomitant Polymorphs in Hg(II) Coordination Polymers

**DOI:** 10.1021/acs.inorgchem.1c03762

**Published:** 2022-03-17

**Authors:** Francisco Sánchez-Férez, Xavier Solans-Monfort, Teresa Calvet, Mercè Font-Bardia, Josefina Pons

**Affiliations:** † Departament de Química, Universitat Autònoma de Barcelona, 08193 Bellaterra, Barcelona, Spain; ‡ Departament de Mineralogia, Petrologia i Geologia Aplicada, Universitat de Barcelona, Martí i Franquès s/n, 08028 Barcelona, Spain; § Unitat de Difracció de Raig-X, Centres Científics i Tecnològics de la Universitat de Barcelona (CCiTUB), Universitat de Barcelona, Solé i Sabarís, 1-3, 08028 Barcelona, Spain

## Abstract

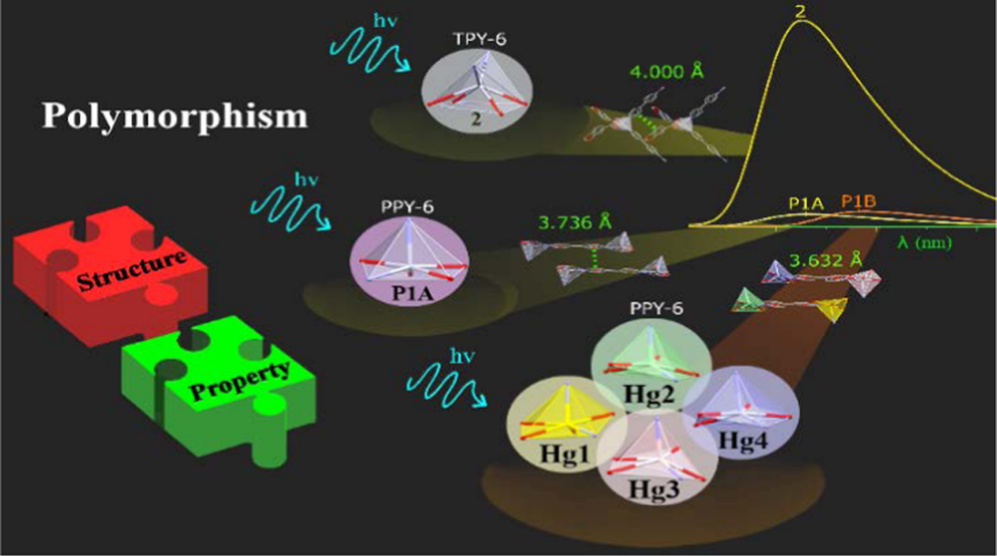

Controlling the formation of the desired product in the appropriate crystalline form is the fundamental breakthrough of crystal engineering. On that basis, the preferential formation between polymorphic forms, which are referred to as different assemblies achieved by changing the disposition or arrangement of the forming units within the crystalline structure, is one of the most challenging topics still to be understood. Herein, we have observed the formation of two concomitant polymorphs with general formula {[Hg(Pip)_2_(4,4′-bipy)]·DMF}*_n_* (**P1A, P1B**; Pip = piperonylic acid; 4,4′-bipy = 4,4′-bipyridine). Besides, [Hg(Pip)_2_(4,4′-bipy)]*_n_* (**2**) has been achieved during the attempts to isolate these polymorphs. The selective synthesis of **P1A** and **P1B** has been successfully achieved by changing the synthetic conditions. The formation of each polymorphic form has been ensured by unit cell measurements and decomposition temperature. The elucidation of their crystal structure revealed **P1A** and **P1B** as polymorphs, which originates from the Hg(II) cores and intermolecular associations, especially pinpointed by Hg···π and π···π interactions. Density functional theory (DFT) calculations suggest that **P1B**, which shows Hg(II) geometries that are further from ideality, is more stable than **P1A** by 13 kJ·mol^–1^ per [Hg(Pip)_2_(4,4′-bipy)]·DMF formula unit, and this larger stability of **P1B** arises mainly from metal···π and π···π interactions between chains. As a result, these structural modifications lead to significant variations of their solid-state photoluminescence.

## Abstract 2

We have successfully isolated two concomitant polymorphs with formula {[Hg(Pip)_2_(4,4′-bipy)]·DMF}*_n_* (**P1A** and **P1B**), as well as their desolvated form **2**. Then, both polymorphs were selectively synthesized by temperature or anion-template formation. Their crystal structures revealed distorted pentagonal pyramidal geometries and show that differences arise from geometry and packing that led to different solid-state photoluminescence emissions. According to periodic-DFT calculations, distortions in **P1B** are counterbalanced leading to a more stable form by Hg(II)···π and π···π interactions.

## Introduction

The rise of crystal engineering, triggered by the identification and understanding of the structural features in materials, enabled the prediction of crystal structures and, therefore, improved the ability to design products with desired chemical and physical properties by tuning their crystal structure.^1,2^ These structural modifications required understanding of molecular and supramolecular preferences and identifying interaction patterns.^3,4^ However, since crystallization is a kinetic process, metastable intermediates can be achieved as multiple local minima during the self-assembly of the ligands with the metal ion.^5^ These structures are mainly stabilized by the steric requirements of the linkers and by hydrogen bonds and noncovalent interactions *inter alia* π···π, C–H···O, C–H···π or the limited case of Hg···π.^6,7^ Therefore, providing access to many potential structure accommodations. Not surprisingly, concomitant polymorphs can be formed within this landscape.^8^

Within this frame, slight modifications, intermediate variations, or major structural changes (polymorphism) throughout the entire crystal structure are promoted by factors such as time,^9^ temperature,^10^ solvent,^11,12^ or the introduction of templates,^13^ which influence both the nucleation rate and crystal growth. While the role of the solvent varies from filling voids and partaking in intermolecular interactions to being coordinated to the metal center,^14^ temperature is a simple way to control the formation of polymorphic forms. Energetic data of organic polymorphs^15^ show that usually energy differences fall within the range of 0–10 kJ·mol^–1^, but scarce data about relative energy calculation of coordination polymers have been found.^16^ Interestingly, these mere differences in the order of weak interactions are responsible for significant modification in the resulting properties. Recent photoluminescence studies of Au(I) coordination complexes have shown emission-dependent properties caused by slight structure differences after the absorption of guest solvent molecules.^13^ By this token, polymorphism in Cu(I) complexes evinced the impact of such structural differences in the photophysical properties.^17^

Although there is a large amount of polymorphism data about crystallization of organic salts^18^ and cocrystals^19^ of pharmaceutical interest, and despite the knowledge of polymorphs in discrete coordination complexes such as the archetypal [Pt(2,2′-bipyridine)Cl_2_],^20^ there is a scarce number of structures bearing coordination polymers reported hitherto comprising Cu(I),^21−23^ Ag(I),^14,24,25^ Co(II) and Ni(II),^26−29^ Cd(II),^16,30^ or Pb(II).^31^ In this scenario, free rotation ligands can drive the formation of conformational polymorphs.^25,28,32,33^ Besides, Hg(II) as softer metal compared to Zn(II) and Cd(II)^34^ is capable of accommodating several distorted geometries and partake in weak intermolecular interactions. Thus, a combination of them can trigger the assembly of different arrangements.^7,35^

In pursuit of extending our knowledge on the structure–property relationship of Hg(II) compounds,^36,37^ we have combined Hg(OAc)_2_, 1,3-benzodioxole-5-carboxylic acid (piperonylic acid, HPip) and the free rotational 4,4′-bipyridine ligand (4,4′-bipy). The synthesis performed at 95 °C resulted in two concomitant polymorphs bearing the same zigzag one-dimensional (1D) structure and accommodating guest *N*,*N*-dimethylformamide (DMF) molecules with general formula {[Hg(Pip)_2_(4,4′-bipy)]·DMF}*_n_* (**P1A** and **P1B**). During the attempts to isolate each polymorphic forms, we recognized the formation of an additional zigzag 1D coordination polymer with formula {[Hg(Pip)_2_(4,4′-bipy)]}*_n_* (**2**). All of them were characterized by analytical and spectroscopic techniques. In addition, periodic density functional theory (DFT) calculations have been performed to set the relative stabilities of both polymorphic forms and rationalize the origin of the different stability. Finally, these structural differences provoked dramatic variations in their solid-state photoluminescence.

## Experimental Section

### Chemical Risks

Hg(II) complexes are toxic, and any manipulation of the samples has to be carried out into the fume hood and wearing gloves.

### Materials and General Details

Hg(II) acetate (Hg(OAc)_2_), 1,3-benzodioxole-5-carboxylic acid (piperonylic acid, HPip), 4,4′-bipyridine (4,4′-bipy) ligands, methanol (MeOH), *N*,*N*-dimethylformamide (DMF), acetic acid (HOAc), and diethyl ether (Et_2_O) as solvents were purchased from Sigma-Aldrich. The water used in the reactions was Milli-Q water. Deuterated dimethyl sulfoxide-*d*_6_ (DMSO-*d*_6_) was used for the NMR experiment and was purchased from Eurisotop. All of them were used without further purification. Reactions and manipulation were carried out in a Digitheat-TFT furnace (JP Selecta) using sealed vials under an autogenous pressure of DMF at 95 °C for the synthesis of the mixture (**P1A** and **P1B**) and the isolation of either **P1A** or **P1B**. Compound **2** was synthesized in DMF at room temperature (RT) or both in MeOH and Milli-Q water at RT and 95 °C. Powder X-ray diffraction (PXRD) patterns were measured with a PANalytical X’Pert PRO MPD θ/θ powder diffractometer of 240 mm radius, in a configuration convergent beam with a focalizing mirror and a transmission geometry with flat samples sandwiched between low-absorbing films. A Cu Kα radiation with λ = 1.5418 Å was used (45 kW and 40 mA). All of them were recorded from 2θ = 5 to 30° with a step scan of 0.0263° and a measuring time of 300 s per step. Thermal decomposition temperature (dT) was measured on a Stuart Melting Point Apparatus SMP30 with a heating ramp of 2.0 °C·min^–1^ in a temperature range of 20–210 °C. Elemental analyses (C, H, N) were carried out on a Euro Vector 3100 instrument. Simultaneous thermogravimetric (TG)/differential thermal analysis (DTA) determinations were performed with a Netzsch STA 409 instrument, using an aluminum oxide powder crucible and an oxide powder as a standard (Al_2_O_3_, PerkinElmer 0419-0197) and heating at 5 °C·min^–1^ from 25 to 350 °C, under nitrogen atmosphere with a flow rate of 80 mL·min^–1^. The Fourier transform infrared-attenuated total reflection (FTIR-ATR) spectra were recorded on a PerkinElmer spectrometer, equipped with a universal attenuated total reflectance (ATR) accessory with diamond window in the range of 4000–500 cm^–1^. ^1^H, ^13^C{^1^H}, and distortionless enhancement by polarization transfer (DEPT)-135 NMR spectra were recorded on an NMR-FT Bruker360 MHz spectrometer in DMSO-*d*_*6*_ solution at RT. All chemical shifts (δ) are given in ppm. Solid-state photoluminescence measurements were recorded using a Varian Cary Eclipse Fluorescence spectrophotometer between 500 and 660 nm. CIE 1931 chromaticity diagram was generated using Origin Pro 2019b software.

#### Synthesis of the Polymorphs Mixture: {[Hg(Pip)_2_(μ-4,4′-bipy)]·DMF}*_n_* (**P1A** and **P1B**)

DMF (2.5 mL) was placed into a 10 mL vial and heated in a furnace until 95 °C. Once the temperature was reached, 4,4′-bipy (44.1 mg, 0.282 mmol) and HPip (94.2 mg, 0.567 mmol) were introduced. When the solution became transparent, Hg(OAc)_2_ (90.1 mg, 0.283) was added and the suspension was sonicated for a minute until dissolution. The reaction was sealed and kept under autogenous pressure at 95 °C for 45 min and then allowed to cool down out of the furnace for 1 h until 25 °C. After cooling, several prism-like colorless crystals were formed, filtered, and washed twice with 5 mL of cold Et_2_O. Careful inspection of them revealed the presence of two different crystal sizes but sharing the same crystalline habit (the smaller ones were around 70%, while the large ones were about 30%). These single crystals were mechanically sorted for their X-ray crystal structure elucidation, revealing that the small crystals were **P1A** while the big crystals were **P1B**.

**P1A** and **P1B**: Yield: 141 mg (66%). dT = 192–196 °C. Anal. Calcd for C_29_H_25_N_3_O_9_Hg (760.11 g·mol^–1^): C, 45.82; H, 3.31; N, 5.53. Found: C, 45.56; H, 3.28; N, 5.32%. FTIR-ATR (wavenumber, cm^–1^): 3097(w)–3014(w) [ν(CH)]_ar_, 2985(w)–2784(w) [ν(CH)]_al_, 1659(m) [ν(C=O)]_DMF_, 1628(w)–1576(m) [ν(C=C/C=N)], 1542(m) [ν(COO)]_as_, 1502(m)–1482(m) [ν(C=C/C=N)], 1429(m) [ν(COO)]_s_, 1418(sh.), 1368(s)–1229(s) [δ(C=C/C=N)], 1163(m), 1128(w), 1101(m), 1069(m), 1034(s) [δ(C–H)]_ip_, 1003(m) [δ(C–H)]_ip_, 936(m), 920(m), 888(m), 866(w), 821(m)–771(s) [δ(C–H)]_oop_, 721(m), 682(m), 660(m), 639(m), 630(m), 582(m), 551(m), 534(m), 503(w). ^1^H NMR (360 MHz; DMSO-*d*_6_; 298 K): δ = 2.72 and 2.88 [6H, s, N–(CH_3_)_2_]_DMF_, 6.10 [4H, s, O–C*H*_2_–O], 6.97 [2H, d, ^3^*J* = 8.1 Hz, O_2_C–C–CH–C*H*], 7.41 [2H, s, O_2_C–C–C*H*–CO], 7.58 [2H, d, ^3^*J* = 8.2 Hz, O_2_C–C–C*H*–CH], 7.92 [4H, d, ^3^*J* = 4.3 Hz, *m*-*H*_py_], 7.94 [1H, s, C*H*O]_DMF_, 8.77 [4H, d, ^3^*J* = 5.3 Hz, *o*-*H*_py_].

#### Synthesis of Compound [Hg(Pip)_2_(μ-4,4′-bipy)]*_n_* (**2**)

To a solution of Hg(OAc)_2_ (100 mg, 0.313 mmol) and HPip (105 mg, 0.631 mmol) in DMF (3 mL), a solution of 4,4′-bipy (49.0 mg, 0.313 mmol) in DMF (2 mL) was added dropwise under vigorous stirring. Immediately, a yellowish solid appeared. The reaction was stirred for 1 h. The solid obtained was filtered and washed with 10 mL of cold MeOH. Suitable crystals were obtained by slow diffusion of 5 mL of MeOH into 1 mL of the mother liquors for 5 days. The phase purity of the sample was confirmed by PXRD.

The synthesis of compound **2** was also achieved at RT and at 95 °C using MeOH or Milli-Q water as a solvent.

Yield: 165 mg (77%). dT = 201–202 °C. Anal. Calcd for C_26_H_18_N_2_O_8_Hg (687.02 g·mol^–1^): C, 45.45; H, 2.64; N, 4.08. Found: C, 45.38; H, 2.38; N, 3.84%. FTIR-ATR (wavenumber, cm^–1^): 3107(w)–3045(w) [ν(CH)]_ar_, 2983(w)–2891(w) [ν(CH)]_al_, 2792(w), 1631(w), 1606(m) [ν(C=C/C=N)], 1574(m), 1541(m) [ν(COO)]_as_, 1496(m) [ν(C=C/C=N)], 1484(m) [ν(C=C/C=N)], 1429(m) [ν(COO)]_s_, 1418(m), 1367(s)–1230(s) [δ(C=C/C=N)], 1160(m), 1106(m), 1094(m), 1067(m), 1034(s) [δ(C–H)]_ip_, 1008(m) [δ(C–H)]_ip_, 930(m), 919(m), 890(m), 818(m)–765(s) [δ(C–H)]_oop_, 721(m), 681(m), 670(m), 633(m), 586(m), 549(m). ^1^H NMR (360 MHz; DMSO-*d*_6_; 298 K): δ = 6.08 [4H, s, O–C*H*_2_–O], 6.95 [2H, d, ^3^*J* = 8.0 Hz, O_2_C–C–CH–C*H*], 7.38 [2H, s, O_2_C–C–C*H*–CO], 7.56 [2H, d, ^3^*J* = 7.9 Hz, O_2_C–C–C*H*–CH], 7.89 [4H, d, ^3^*J* = 4.3 Hz, *m*-*H*_py_], 8.74 [4H, d, ^3^*J* = 4.3 Hz, *o*-*H*_py_]. ^13^C{^1^H} NMR (360 MHz; DMSO-*d*_6_; 298 K): δ = 169.07 [O_2_*C*–C], 150.63 [N–*C*H–CH], 150.13 [O_2_C–C–(CH)_2_–*C*], 147.13 [O_2_C–C–CH–*C*], 144.95 [N–(CH)_2_–*C*], 127.64 [O_2_C–*C*], 125.17 [O_2_C–C–*C*H–CH], 121.97 [N–CH–*C*H], 109.63 [O_2_C–C–*C*H–C], 107.81 [O_2_C–C–CH–*C*H], 101.66 [O–*C*H_2_–O].

##### Synthesis of {[Hg(Pip)_2_(μ-4,4′-bipy)]·DMF}*_n_* (**P1A**)

DMF (5 mL) and HOAc (10.9 μL, 0.190 mmol) were introduced into a 10 mL vial, sealed, and heated in a furnace until 95 °C. Once the temperature was reached, compound **2** (65.3 mg, 0.0950 mmol) was added and the mixture was sonicated for 1 min until a yellowish transparent solution was obtained. Then, the vial was sealed, kept at 95 °C under an autogenous pressure for 45 min, and left to cool down slowly for 7 h until 25 °C (Figure S1, Supporting Information). Suitable crystals of **P1A** were formed, collected, filtered off, and washed twice with 5 mL of cold Et_2_O.

Yield: 42.8 mg (59%). dT = 192–193 °C. FTIR-ATR (wavenumber, cm^–1^): 3098(w)–3012(w) [ν(CH)]_ar_, 2895(w) [ν(CH)]_al_, 2780(w) [ν(CH)]_al_, 1658(s) [ν(C=O)]_DMF_, 1628(w)–1576(m) [ν(C=C/C=N)], 1542(m) [ν(COO)]_as_, 1503(m) [ν(C=C/C=N)], 1483(m) [ν(C=C/C=N)], 1427(m) [ν(COO)]_s_, 1368(s)–1229(s) [δ(C=C/C=N)], 1163(m), 1101(m), 1069(m), 1035(s) [δ(C–H)]_ip_, 1003(sh), 935(m), 888(m), 861(w), 820(m)–771(s) [δ(C–H)]_oop_, 719(m), 680(m), 660(m), 639(m), 629(m), 582(m), 550(m), 538(m), 511(m).

##### Synthesis of {[Hg(Pip)_2_(μ-4,4′-bipy)]·DMF}*_n_* (**P1B**)

The synthesis of **P1B** was performed following the same procedure as in the synthesis of **P1A** but without adding HOAc in the recrystallization of **2** (80.0 mg, 0.117 mmol).

Yield: 64.9 mg (73%). dT = 198–199 °C. 3098(w)–3012(w) [ν(CH)]_ar_, 2894(w) [ν(CH)]_al_, 2786(w) [ν(CH)]_al_, 1658(s) [ν(C=O)]_DMF_, 1628(w)–1578(m) [ν(C=C/C=N)], 1540(m) [ν(COO)]_as_, 1504(m)–1453(m) [ν(C=C/C=N)], 1428(m) [ν(COO)]_s_, 1419(m), 1367(s)–1230(s) [δ(C=C/C=N)], 1163(m), 1101(m), 1070(m), 1036(s) [δ(C–H)]_ip_, 1006(sh), 935(m), 920(m), 887(m), 866(w), 819(m)–769(s) [δ(C–H)]_oop_, 720(m), 680(m), 661(m), 637(m), 630(m), 582(m), 554(m), 536(m).

### X-ray Crystallographic Data and Structural Analysis

Colorless prism-like **P1A**, **P1B**, and **2** specimens were used for the X-ray crystallographic analysis. The X-ray intensity data were measured on a D8 Venture system equipped with a multilayer monochromator and a Mo microfocus (λ = 0.71073 Å). For **P1A**, **P1B**, and **2** the frames were integrated with the Bruker SAINT software package, using a narrow-frame algorithm. For **P1A**, the integration of the data using a triclinic unit cell yielded a total of 8231 reflections to a maximum θ value of 30.60° (0.70 Å resolution), of which 8231 were independent (average redundancy 1.000, completeness = 99.0%, *R*_int_ = 18.28%, *R*_sig_ = 17.88%) and 3761 (45.69%) were greater than 2σ(|*F*|^2^). For **P1B**, the integration of the data using a triclinic unit cell yielded a total of 216 059 reflections to a maximum θ value of 30.55° (0.70 Å resolution), of which 33 014 were independent (average redundancy 6.544, completeness = 99.9%, *R*_int_ = 5.74%, *R*_sig_ = 3.96%) and 26 560 (80.45%) were greater than 2σ(|*F*|^2^). For **2**, the integration of the data using a monoclinic unit cell yielded a total of 3368 reflections to a maximum θ value of 30.36° (0.70 Å resolution), of which 3368 were independent (average redundancy 1.000, completeness = 97.3%, *R*_int_ = 4.05, *R*_sig_ = 2.99%) and 3237 (96.11%) were greater than 2σ(|*F*|^2^).

The structures were solved and refined using the Bruker SHELXTL Software Package (version-2018/3).^38^ For **P1A**, the final anisotropic full-matrix least-squares refinement on |*F*|^2^ with 330 variables converged at *R*_1_ = 4.58%, for the observed data and w*R*_2_ = 12.50% for all data. For **P1B**, the final anisotropic full-matrix least-squares refinement on |*F*|^2^ with 1556 variables converged at *R*_1_ = 2.95%, for the observed data and w*R*_2_ = 6.80% for all data. For **2**, the final anisotropic full-matrix least-squares refinement on |*F*|^2^ with 168 variables converged at *R*_1_ = 2.18%, for the observed data and w*R*_2_ = 5.43% for all data. For **P1A**, **P1B**, and **2**, the final cell constants and volume are based upon the refinement of the *XYZ*-centroids of reflections above 20 σ(*I*). Data were corrected for absorption effects using the multiscan method (SADABS). Crystal data and relevant details of structure refinement for compounds **P1A**, **P1B**, and **2** are reported in Table 1. Selected bond lengths, angles, and intermolecular interactions of **P1A** are listed in Table 2. Bond lengths, angles, and intermolecular interactions of **P1B** are shown in Tables 3–5, respectively. Bond lengths, angles, and intermolecular interactions of **2** are listed in Table 6.

**Table 1:** Crystal Structure Refinement Data for Compounds **P1A**, **P1B**, and **2**

	**P1A**	**P1B**	**2**
empirical formula	C_29_H_25_HgN_3_O_9_	C_116_H_100_Hg_4_N_12_O_36_	C_26_H_18_HgN_2_O_8_
formula weight	760.11	3040.43	687.01
*T* (K)	100(2)	100(2)	100(2)
wavelength (Å)	0.71073	0.71073	0.71073
system, space group	triclinic, *P*1̅	triclinic, *P*1̅	monoclinic, *C*2/*c*
unit cell dimensions			
*a* (Å)	6.0027(7)	13.6326(15)	17.803(6)
*b* (Å)	13.5859(17)	20.267(2)	11.556(4)
*c* (Å)	17.769(2)	21.899(2)	12.339(4)
α (deg)	71.181(4)	105.796(4)	90
β (deg)	83.249(4)	105.114(4)	115.059(14)
γ (deg)	80.217(4)	100.923(4)	90
*V* (Å^3^)	1348.6(3)	5395.3(10)	2299.5(13)
*Z*	2	2	4
*D*_calc_ (g·cm^3^)	1.872	1.872	1.984
μ (mm^–1^)	5.769	5.768	6.751
*F*(000)	744	2976	1328
crystal size (mm^3^)	0.250 × 0.105 × 0.045	0.303 × 0.177 × 0.060	0.701 × 0.198 × 0.148
*hkl* ranges	–8 ≤ *h* ≤ 8	–19 ≤ *h* ≤ 19	–25 ≤ *h* ≤ 22
	–18 ≤ *k* ≤ 19	–28 ≤ *k* ≤ 28	0 ≤ *k* ≤ 16
	0 ≤ *l* ≤ 25	–31 ≤ *l* ≤ 31	0 ≤ *l* ≤ 17
2θ range (deg)	2.427–30.602	1.966–30.551	3.526–30.365
reflections collected/unique/[*R*_int_]	8230/8230	216 059/33 014	3368/3368
	[*R*_int_] = 0.1828	[*R*_int_] = 0.0574	[*R*_int_] = 0.0405
completeness to θ (%)	99.9	99.9	97.8
absorption correction	semiempirical	semiempirical	semiempirical
max. and min. transmis.	0.7461 and 0.5741	0.7461 and 0.5645	0.7461 and 0.3631
refinement method	full-matrix least-squares on |*F*|^2^	full-matrix least-squares on |*F*|^2^	full-matrix least-squares on |*F*|^2^
data/restrains/parameters	8230/1/330	33 014/0/1556	3368/0/168
goodness of fit (GOF) on |*F|*^2^	0.923	1.061	1.043
final *R* indices [*I* > 2σ(*I*)]	*R*_1_ = 0.0458, w*R*_2_ = 0.0924	*R*_1_ = 0.0295, w*R*_2_ = 0.0579	*R*_1_ = 0.0218, w*R*_2_ = 0.0534
*R* indices (all data)	*R*_1_ = 0.1549, w*R*_2_ = 0.1250	*R*_1_ = 0.0482, w*R*_2_ = 0.0680	*R*_1_ = 0.0229, w*R*_2_ = 0.0543
extinction coefficient	0.0027(3)	n/a	n/a
largest. diff. peak and hole (e·Å^–3^)	1.807 and −1.841	2.418 and −1.894	2.128 and −1.765

**Table 2:** Bond Lengths (Å), Bond and Torsion Angles (deg), and Intermolecular Interactions Present in **P1A**^a^

bond lengths					
Hg(1)–O(1)	2.192(5)	Hg(1)–O(2)	2.901(9)	Hg(1)–O(2)	2.901(9)
Hg(1)–O(5)	2.228(9)	Hg(1)–O(6)	2.647(9)	Hg(1)–O(6)	2.647(9)
Hg(1)–N(1)	2.215(6)	Hg(1)–N(2)	2.341(6)	Hg(1)–N(2)	2.341(6)

bond angles					
N(2)–Hg(1)–O(1)	91.0(2)	N(1)–Hg(1)–O(6)	85.3(3)	N(1)–Hg(1)–O(6)	85.3(3)
N(2)–Hg(1)–O(2)	101.7(2)	O(1)–Hg(1)–O(2)	50.1(2)	O(1)–Hg(1)–O(2)	50.1(2)
N(2)–Hg(1)–O(5)	102.9(3)	O(1)–Hg(1)–O(5)	95.5(3)	O(1)–Hg(1)–O(5)	95.5(3)
N(2)–Hg(1)–O(6)	84.1(3)	O(1)–Hg(1)–O(6)	141.7(3)	O(1)–Hg(1)–O(6)	141.7(3)
N(2)–Hg(1)–N(1)	104.3(2)	O(2)–Hg(1)–O(5)	137.6(3)	O(2)–Hg(1)–O(5)	137.6(3)
N(1)–Hg(1)–O(2)	82.5(2)	O(2)–Hg(1)–O(6)	167.5(3)	O(2)–Hg(1)–O(6)	167.5(3)
N(1)–Hg(1)–O(1)	132.4(2)	O(5)–Hg(1)–O(6)	49.5(4)	O(5)–Hg(1)–O(6)	49.5(4)
N(1)–Hg(1)–O(5)	123.3(3)				

Cg(*I*)–Cg(*J*)	torsion angle, χ
Cg(1)–Cg(1)	1.8(11)

intermolecular interactions	H···A (Å)	D···A (Å)	D–H (Å)	>D–H···A (deg)
C(15)–H(15)···O(9)	2.318	3.229(9)	0.950	160.4
C(17)–H(17)···O(9)	2.441	3.340(10)	0.950	158.3

π interactions			
Cg(*I*)···Hg(*J*)	Cg···Hg^b^	MeJ_Perp	β
Cg(1)···Hg(1)	3.953	3.491	27.91

Cg(*I*)···Cg(*J*)	Cg···Cg^c^	α^d^	β, γ^e^	Cg(*I*)_Perp, Cg(*J*)_Perp^f^	slippage^g^
Cg(1)···Cg(1)	3.736(5)	0.0(4)	22.8, 22.8	3.443(3)	1.449

a Cg···Cg and Cg···Hg distances are given in Å.

b Cg···Hg = distance between ring centroid and Hg(II) center.

c Cg···Cg = distance between ring centroids (Å).

d α = dihedral angle between planes I and J (deg).

e Offset angles: β = angle Cg(*I*)–Cg(*J*) and normal to plane *I* (deg) and γ = angle Cg(*I*)–Cg(*J*) and normal to plane *J* (deg) (β = γ, when α = 0).

f Perpendicular distance (Å) of Cg(*I*) on plane *J* and perpendicular distance (Å) of Cg(*J*) on plane *I* (equal when α = 0).

g Slippage = horizontal displacement or slippage between Cg(*I*) and Cg(*J*) (equal for both centroids when α = 0). Cg(1) = N1–C9–C10–C11–C12–C13.

**Table 3:** Bond Lengths (Å) and Torsion Angles (deg) of **P1B**^a^

bond lengths			
Hg(1)			
Hg(1)–O(1)	2.210(2)	Hg(1)–O(2)	2.964(3)
Hg(1)–O(5)	2.270(2)	Hg(1)–O(6)	2.732(3)
Hg(1)–N(1)	2.350(3)	Hg(1)–N(2)	2.192(3)
Hg(2)			
Hg(2)–O(13)	2.212(2)	Hg(2)–O(14)	2.916(3)
Hg(2)–O(9)	2.274(2)	Hg(2)–O(10)	2.728(3)
Hg(2)–N(3)	2.213(3)	Hg(2)–N(4)	2.363(3)
Hg(3)			
Hg(3)–O(17)	2.216(2)	Hg(3)–O(18)	2.885(3)
Hg(3)–O(21)	2.260(2)	Hg(3)–O(22)	2.740(3)
Hg(3)–N(5)	2.376(3)	Hg(3)–N(6)	2.212(3)
Hg(4)			
Hg(4)–O(29)	2.245(2)	Hg(4)–O(30)	2.794(3)
Hg(4)–O(25)	2.267(2)	Hg(4)–O(26)	2.700(3)
Hg(4)–N(7)	2.237(3)	Hg(4)–N(8)	2.326(3)

Cg(*I*)–Cg(*J*)	torsion angle, χ	Cg(*I*)–Cg(*J*)	torsion angle, χ
Cg(1)–Cg(3)	8.0(5)	Cg(5)–Cg(6)	3.5(5)
Cg(2)–Cg(4)	34.4(5)	Cg(7)–Cg(7)	0.1(6)

a Cg(1) = N2–C22–C23–C24–C25–C26; Cg(2) = N6–C74–C75C76–C77–C78; Cg(3) = N3–C27–C28–C29–C30–C31; Cg(4) = N7–C79–C80–C81–C82–C83; Cg(5) = N4–C48–C49–C50–C51–C52; Cg(6) = N5–C53–C54–C55–C56–C57; Cg(7) = N8–C100–C101–C102–C103–C104.

The geometry evaluation of the three complexes has been performed using version 2.1 of SHAPE^39^ software, which is based on the low continuous-shape measure (CShM) value *S.*(40) The corresponding atomic coordinates have been directly extracted from the .cif data. Hirshfeld surfaces with their 2D fingerprint plots have been generated using CrystalExplorer 17.5.^41^ The essential interactions have been divided into O···H/H···O, C···C and those involving Hg(II).

Complete information about the crystal structure and molecular geometry is available in .cif format and deposited in the CCDC. CCDC numbers 2101056–2101058 contain the supporting data of this paper. Molecular graphics were generated using Mercury (version 4.3.1)^42^ with POV-Ray Package (version 3.7).^43^ Color codes for molecular graphics: light slate blue (N), suva gray (C), white (H), lavender gray (Hg), and red (O).

### Computational Details

DFT calculations of **P1A** and **P1B** were carried out using the VASP code.^44,45^ Geometry optimizations were performed at the PBE-D2 level of theory^46,47^ assuming a P1 space group and keeping the unit cell parameters to those determined by single-crystal X-ray diffraction (SC-XRD). With the aim of analyzing the suitability of the D2 Grimme’s empirical correction, we compared the relative stability of **P1B** and **P1A** (in terms of potential energy per [Hg(Pip)_2_(4,4′-bipy)]·DMF unit formula) with PBE-D2, PBE-D3 and PBE-D*, a modification of the D2 Grimme’s empirical term suggested to be more suitable for the modeling of molecular crystals.^48^ Results are reported in Table S1 in the Supporting Information and show that all Grimme’s correction leads to a preference for the **P1B** polymorph and the differences between the three methods are small (5.5 kJ·mol^–1^). Moreover, according to the literature, PBE-D3 is accurate enough for modeling molecular crystals^49,50^ and, indeed, it has been even used to compute reference values for the validation of less computationally demanding approaches.^51^ Ionic cores were described with the projector augmented wave (PAW) pseudopotentials.^52,53^ The valence electrons were represented through a plane-wave basis set with a kinetic energy cutoff of 600 eV. Since cell size and the number of Hg(II) units in the cell differs for **P1A** and **P1B**, the Brillouin zone was sampled with a different Monkhorst–Pack *K*-point mesh in each case, *i.e.*, (4 × 4 × 4) and (1 × 1 × 1) for **P1A** and **P1B**, respectively.

With the aim of getting further insights into the relative stability of the two polymorphs, we performed an energy partitioning scheme decomposing the association energy (*E*) between two metal dimers of vicinal chains in two terms: (i) the energy cost to distort the dimers from the optimal geometry of a hypothetical isolated fragment (*E*_DIS_) and (ii) the pure interaction energy (*E*_INT_) between the already distorted fragments (eq 1).
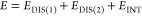
1

These energy partition schemes are standard in computational chemistry and examples where they have been applied with success can be found in the literature.^54,55^ This analysis was made with molecular calculations that were performed at the same PBE-D2 level of theory and using Gaussian16 package.^56^ Main group elements and valence electrons of Hg(II) were represented with the Pople 6-31+G(d,p)^57,58^ and Dunning’s aug-cc-pVDZ^59^ Gaussian-type basis sets, respectively. The Hg(II) core was taken into account with the pseudopotential associated with aug-cc-pVDZ basis set.^60^

## Results and Discussion

### Formation of **P1A**, **P1B**, and **2**

**Scheme 1:**
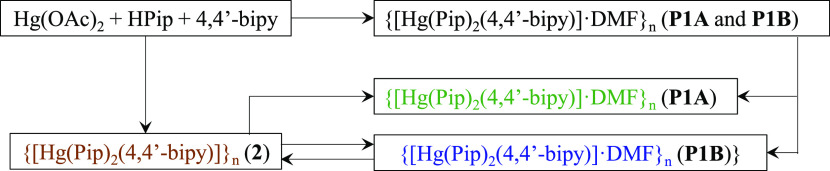
Outline of the Formation of the Mixture (**P1A** and **P1B**) and **2** and the Isolation of **P1A** and **P1B**

From the reaction of Hg(OAc)_2_, HPip and 4,4′-bipy in DMF as a solvent, three different crystalline products, namely, {[Hg(Pip)_2_(μ-4,4′-bipy)]·DMF}*_n_* (**P1A** and **P1B**) and [Hg(Pip)_2_(μ-4,4′-bipy)]*_n_* (**2**) have been isolated (Scheme 1) depending on the synthetic conditions.

The concomitant crystallization of **P1A** and **P1B** was observed when the reaction was performed in DMF at 95 °C. Cooling down of the saturated solution for 1 h resulted in a crystals mixture of **P1A** and **P1B**. The main difference between both crystals at first sight is the size, being **P1B** larger than **P1A**. Instead, the formation of **2** is achieved at RT in DMF, MeOH, and Milli-Q water or also at 95 °C in MeOH and Milli-Q water. The different formation of the mixture **P1A** and **P1B** or **2** could rely on the different solubility of the final complexes under the reaction conditions. Compound **2** rapidly precipitates as a yellow powder when the reaction is performed at RT in DMF, while it is soluble at 95 °C. We have performed the reaction in a temperature range of 25–115 °C, once every 10 °C and the complete dissolution and formation of the polymorphs is only observed as of 75 °C and rapidly precipitates as temperature drops. Over 105 °C, complexes start to decompose. Their low solubility seems to facilitate the crystallization of different polymorphic intermediates, which suddenly nucleated and grew as temperature dropped.^9^ Therefore, temperature and thereby solubility are the key factors to the formation of the polymorphic mixture.

**Scheme 2:**
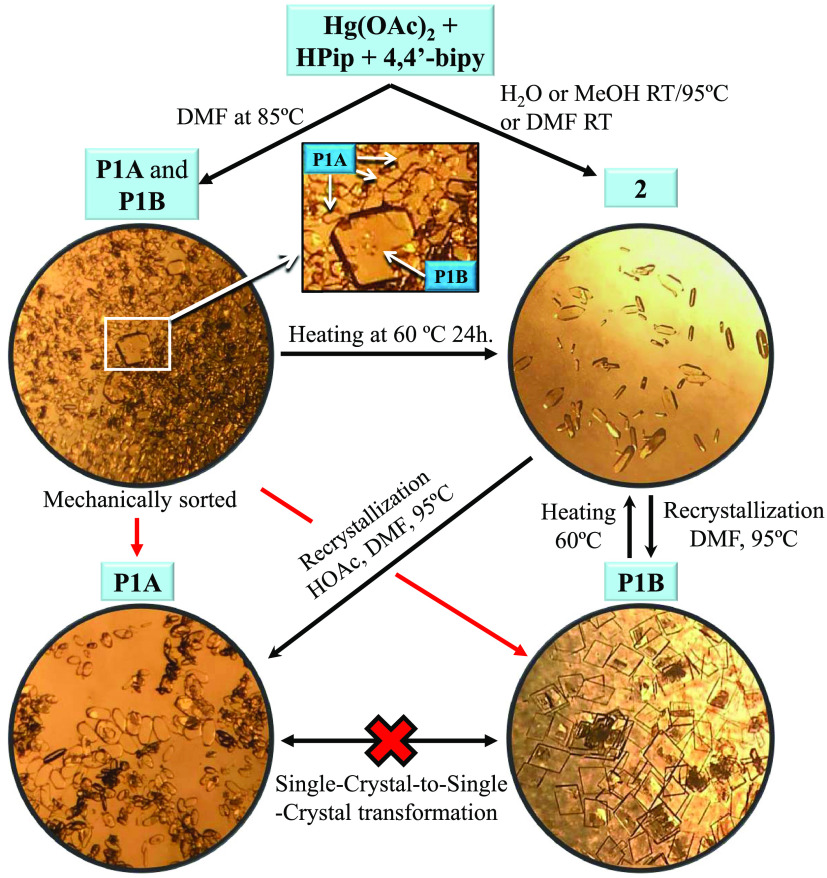
Optical Microphotographs of Single Crystals of **P1A**, **P1B**, and **2** from the Synthesis of the Mixture or Achieved by Recrystallization of **2** Inset: **P1A** and **P1B** Mixture.

The isolation of polymorphic forms being initially found to concomitantly crystallize as a mixture has been one of the pillars of crystal engineering. However, to attain the proper conditions to reach the target crystal form is often intricated. In this scenario, one of the forms usually tends to be less stable and, hence, more complicated to be achieved.^16^ The ability of the linkers to arrange into different polymorphic forms relies on directing factors *inter alia* time, temperature, concentration, solvent, or additional anions during the crystallization step.^12^ Therefore, we modified the synthetic conditions trying to isolate **P1A** and **P1B**. These polymorphs were synthesized as single crystals by changing concentration, time, and temperature or adding HOAc to identify the formation of the polymorphs. After the initially found concomitant formation of **P1A** and **P1B** (Scheme 2), the isolation of **P1B** has been accomplished by recrystallization of **2** in DMF at 95 °C and slow cooling down for 7 h. The recrystallization of **2** to achieve **P1B** has been performed in a concentration range of 1.1 × 10^–3^–2.5 × 10^–2^ M, from which the optimal conditions to crystallize **P1B** were found to be between 1.0 × 10^–2^ and 2.5 × 10^–2^ M. It is worth mentioning that **P1B** was formed regardless of the concentration while the formation of **P1A** was not observed. Since the initial reaction starting from Hg(OAc)_2_ resulted in the mixture of **P1A** and **P1B**, with **P1A** being the predominant form, we added equivalent amounts of HOAc to incorporate OAc^–^ anions during the recrystallization of **2** in DMF at 95 °C. Interestingly, the addition of the OAc^–^ anions drove the formation of **P1A** (Scheme 2). All of those syntheses were examined by several single-crystal X-ray diffraction analyses to determine their unit cell parameters, which are markedly different between **P1A** and **P1B**. In addition, the unit cell of crystals previously measured was redetermined after up to 3 weeks and no interconversion between **P1A** and **P1B** was observed.

**Figure 1:**
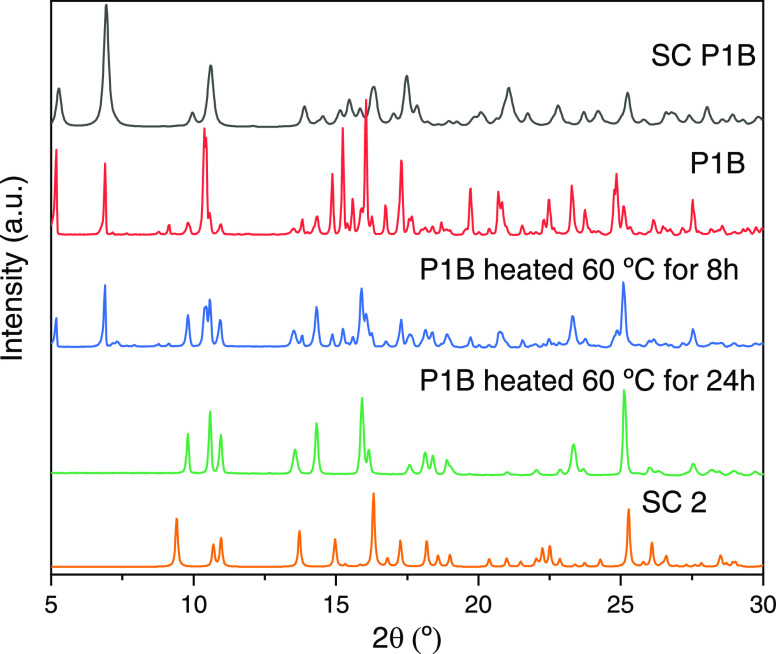
Comparative diffractograms from top to bottom: single-crystal (SC) XRD pattern of **P1B**, PXRD of bulk **P1B**, PXRD of **P1B** after heating at 60 °C for 8 h, PXRD of bulk **P1B** after heating at 60 °C for 24 h, and SC-XRD of **2**.

Some of the unit cell parameters measured are listed in Table S2, Supporting Information. The formation of each polymorphic form has been traced by single-crystal X-ray diffraction (SC-XRD), and the phase purity of **2** was confirmed by powder X-ray diffraction (PXRD) (Figure S2, Supporting Information). We also provide the PXRD pattern of **P1B**, confirmed by unit cell measurements, to ensure the absence of **2** in the sample (Figure S3, Supporting Information). The interconversion between **P1B** and **2** was followed by PXRD (Figure 1).

### General Characterization

The three compounds were characterized by decomposition temperature (dT), elemental analysis (EA), FTIR-ATR and ^1^H NMR spectroscopies, TG/DTA, and single-crystal X-ray diffraction. Compound **2** was characterized by ^13^C{^1^H} and DEPT-135 NMR spectroscopies. In addition, the solid-state photoluminescence of the three complexes has been recorded.

We have recorded the FTIR-ATR spectra of **P1A** and **P1B**, **P1A**, **P1B**, and **2**. The FTIR-ATR spectrum of the mixture of **P1A** and **P1B** is a combination of the spectra of the isolated products. The absence of bands in all of the FTIR-ATR spectra between 2630 and 2518 cm^–1^, attributable to hydrogen-bonded ν(O–H)_HPip_ and at 1667 cm^–1^ from ν(C=O)_HPip_, indicates the deprotonation of the HPip ligand. The corresponding carboxylate bands appear at 1542 cm^–1^ (**P1A**), 1540 cm^–1^ (**P1B**), or 1560 cm^–1^ (**2**) for ν_as_(COO) and at 1427 cm^–1^ (**P1A**), 1428 cm^–1^ (**P1B**), or 1430 cm^–1^ (**2**) for ν_s_(COO) (Figures S4–S9, Supporting Information). The difference between these bands (Δ = ν_as_(COO) – ν_s_(COO)) reveals the coordination modes of the carboxylate linkers:^61^ 115 cm^–1^ (**P1A**), 112 cm^–1^ (**P1B**), and 130 cm^–1^ (**2**), suggesting bidentate chelate (μ_1_–η^2^) coordination mode in the three compounds. In addition, the spectra of **P1A** and **P1B** show a characteristic peak at 1658 cm^–1^ corresponding to the ν(C=O)_DMF_.^62^^62^ Additional bands from the aromatic rings have also been identified.^63,64^

The ^1^H NMR spectra of **P1A** and **P1B** mixture and **2** have been recorded in DMSO-*d*_6_ (Figures S10 and S11, Supporting Information). In the mixture of **P1A** and **P1B**, the presence of DMF is confirmed by signals at 7.94, 2.88, and 2.72 ppm. The spectra show aromatic signals from the Pip linkers between 7.58 and 6.95 ppm and the −C*H*_2_– from the dioxole group at 6.10 ppm (**P1A** + **P1B**) and 6.08 ppm (**2**). The two signals from the 4,4′-bipy appear between 8.77 and 7.89 ppm. The ^13^C{^1^H} and DEPT-135 NMR spectra of **2** display the carboxylate band at 169.07 ppm and the −*CH*_*2*_– of the dioxole at 101.66 ppm. The remaining *C* signals appear between 150.63 and 108.81 ppm. DEPT-135 experiment was required to ensure the correct assignation of the carbon atoms from the aromatic rings (Figure S12, Supporting Information).

TG–DTA determinations of the complexes were performed using 79.9 mg (**P1A**), 46.8 mg (**P1B**), or 81.2 mg (**2**). The TG analysis of **2** exhibits a flat profile without any mass loss up to 187 °C. Besides, its decomposition temperature is set to 199 °C from the DTA data (Figure S13, Supporting Information). The TG analysis of **P1A** and **P1B** evidences the loss of a DMF molecule (**P1A**, exp. 7.30%; calcd 9.60%; **P1B** exp. 8.60%; calcd 9.60%) between 50 and 121 °C (**P1A**) or between 90 and 126 °C (**P1B**), and no more thermal events were observed until decomposition, being stable up to 193 °C (**P1A**) or 199 °C (**P1B**). From these data, it seems that the release of DMF by heating is favored in **P1A** with respect to **P1B** (Figures S14 and S15, Supporting Information).

### Interconversion between **P1A**, **P1B**, and **2**

All of the attempts to convert **P1A** into **P1B** and *vice versa* by applying temperature, unavoidably ended in their transformation into **2**. Single crystals of **P1A** and **P1B** are stable under air exposure and no interconversion over time was observed. PXRD confirmed that in solid state, a single-crystal-to-single-crystal transformation between **P1B** and **2** gradually occurs upon heating at 60 °C by losing the occluded DMF molecules (Figure 1).

**Figure 2:**
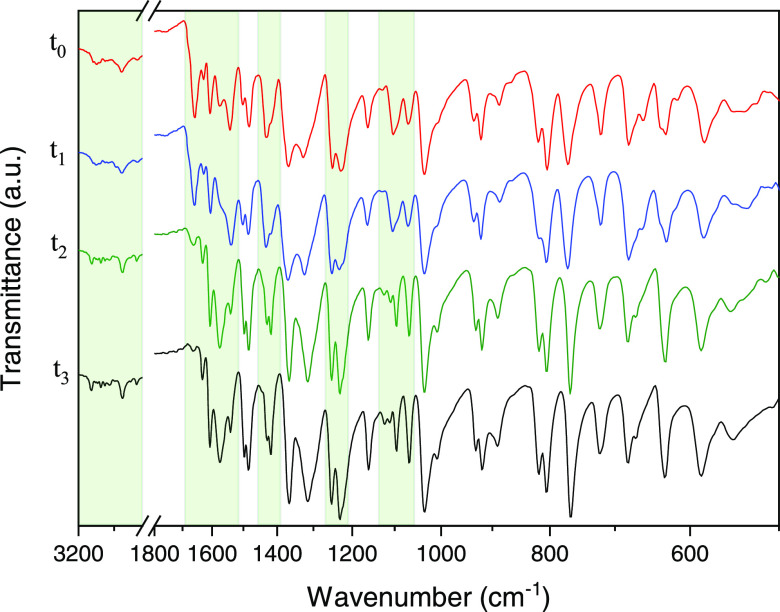
Time-dependent FTIR-ATR spectra of **P1B** as of heating at 60 °C. From top to bottom, *t*_0_ = as-synthesized **P1B**; *t*_1_ = after 1 h 30 min; *t*_2_ = after 12 h; *t*_3_ = after 19 h. The regions in which significant variations of the spectra occur have been highlighted in light green.

Likewise, FTIR-ATR spectra of **P1B** evinced the loss of DMF molecules by the disappearance of the band at 1659 cm^–1^ attributed to the υ(C=O)_DMF_ (Figure 2). Once the transformation into **2** is achieved, the sample can be recrystallized using the conditions mentioned in the Experimental Section to synthesize single crystals of **P1A** or **P1B**. Therefore, polymorphs **P1A** and **P1B** seem to be monotropically related, and interconversion between them is only feasible *via* the formation of **2**, which is promoted by the loss of the DMF molecules and the consequent structural reorganization ending in **2**.

### Crystal Structures of **P1A**, **P1B**, and **2**

**Figure 3:**
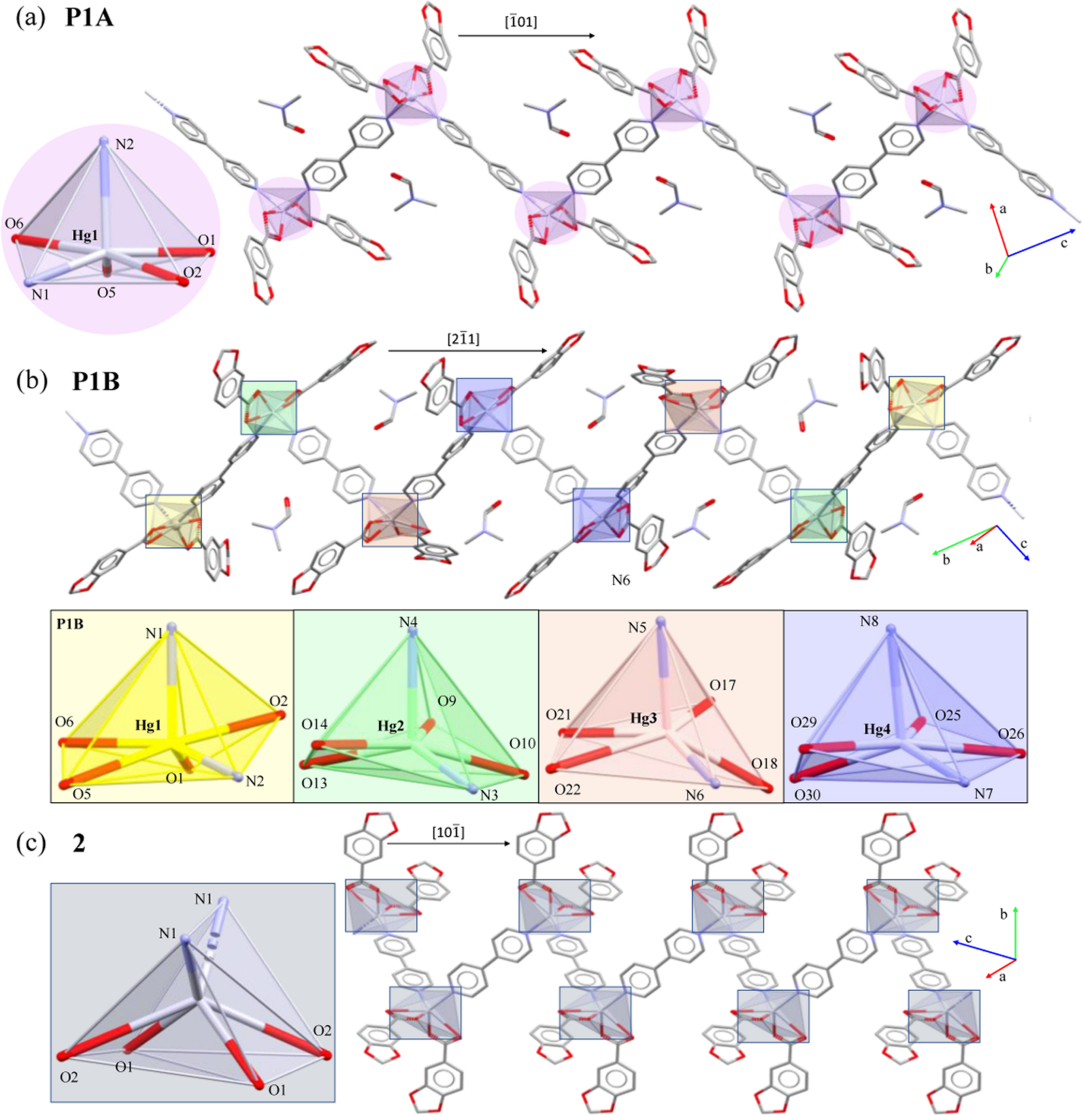
One-dimensional polymeric chains of (a) **P1A**, (b) **P1B**, and (c) **2**. Insets: sequence of the different Hg(II) *cores* for each compound.

Both polymorphs (**P1A** and **P1B**) crystallize in the triclinic *P*1̅ space group, whereas **2** crystallizes in the monoclinic *C*2/*c* space group. All of them present the same connectivity forming zigzag 1D polymeric structures, in which hexacoordinated Hg(II) centers bearing [HgO_4_N_2_] *cores* are assembled by bridging 4,4′-bipy ligands along the [1̅01] (**P1A**) (Figure 3a), [21̅1] (**P1B**) (Figure 3b), or [101̅] (**2**) (Figure 3c) directions. The geometry of **P1A** and **P1B** is of distorted pentagonal pyramid (PPY-6), a seldom reported geometry present in a few complexes and reserved to Cd^65^ or Hg^66^ when they are coordinated to linkers forcing geometric constrains. The geometric distortions have been evaluated by the low continuous-shape measure (CShM) value *S*(39,40) for the three potential geometries comprised in coordination number 6 (Table S3, Supporting Information). The resulting *S* values agree with the better fitting of **P1A** (*S* = 5.748) and **P1B** (*S*: 7.550, Hg1; 6.450, Hg2; 7.466, Hg3; 5.863, Hg4) with a PPY-6 and the pairing of **2** with a trigonal prismatic geometry (TPY-6, *S* = 8.452). **P1A** is assembled by planar 4,4′-bipy linkers (Cg(1)–Cg(1); torsion angle, χ = 1.8(11)°) (Table 2), while **P1B** contains four Hg(II) centers connected by 4,4′-bipy ligands oriented with a χ up to 34.4(5)° (Cg(1)–Cg(3), χ = 8.0(5)°; Cg(5)–Cg(6), 3.5(5)°; Cg(2)–Cg(4), 34.4(5)°; Cg(7)–Cg(7), 0.1(6)°) (Table 3). Compound **2** and **P1A** only have a single Cg(1)–Cg(1) χ value of 1.8(11)° (**P1A**) and 0.1(3)° (**2**).

Such an unusual geometry of the Hg (II) centers in the structure of **P1A** and **P1B** seems to be supported by the assembly of the polymeric chains in columns of stacked and coplanar 4,4′-bipy ligands. The cooperative π···π and Hg···π interactions between the stacked 4,4′-bipy bring the chains closer, thus promoting the displacement of the carboxylate oxygen atoms from the Pip ligands toward the basal plane. The three complexes present similar bond lengths but markedly different bond angles. This is emphasized in the N–Hg–N bond angle being larger in **P1A** (104.3(2)°, Table 2) and **P1B** (104.2(1)–105.2(1)°, Table 4) with respect to **2** (89.82(10)°, Table 6).

**Table 4:** Bond Angles (deg) of **P1B**

bond angles			
Hg(1)			
N(2)–Hg(1)–O(1)	133.44(9)	N(1)–Hg(1)–O(6)	80.80(9)
N(2)–Hg(1)–O(2)	84.86(9)	O(1)–Hg(1)–O(2)	48.60(8)
N(2)–Hg(1)–O(5)	122.70(9)	O(1)–Hg(1)–O(5)	92.68(9)
N(2)–Hg(1)–O(6)	89.53(9)	O(1)–Hg(1)–O(6)	136.85(9)
N(2)–Hg(1)–N(1)	104.53(9)	O(2)–Hg(1)–O(5)	129.76(8)
N(1)–Hg(1)–O(2)	103.18(9)	O(2)–Hg(1)–O(6)	173.79(8)
N(1)–Hg(1)–O(1)	89.83(9)	O(5)–Hg(1)–O(6)	51.95(8)
N(1)–Hg(1)–O(5)	107.84(9)		
Hg(2)			
N(3)–Hg(2)–O(13)	132.30(10)	N(4)–Hg(2)–O(10)	82.42(9)
N(3)–Hg(2)–O(14)	82.98(9)	O(13)–Hg(2)–O(14)	49.39(8)
N(3)–Hg(2)–O(9)	123.80(10)	O(13)–Hg(2)–O(9)	94.7(1)
N(3)–Hg(2)–O(10)	84.82(9)	O(13)–Hg(2)–O(10)	142.50(9)
N(3)–Hg(2)–N(4)	105.20(10)	O(14)–Hg(2)–O(9)	130.69(9)
N(4)–Hg(2)–O(14)	109.42(9)	O(14)–Hg(2)–O(10)	164.88(8)
N(4)–Hg(2)–O(13)	90.70(10)	O(9)–Hg(2)–O(10)	51.72(9)
N(4)–Hg(2)–O(9)	102.30(10)		
Hg(3)			
N(6)–Hg(3)–O(17)	129.80(10)	N(5)–Hg(3)–O(22)	126.59(9)
N(6)–Hg(3)–O(18)	81.92(9)	O(17)–Hg(3)–O(18)	49.83(9)
N(6)–Hg(3)–O(21)	129.00(10)	O(17)–Hg(3)–O(21)	96.70(10)
N(6)–Hg(3)–O(22)	81.52(9)	O(17)–Hg(3)–O(22)	124.63(9)
N(6)–Hg(3)–N(5)	104.60(10)	O(18)–Hg(3)–O(21)	146.43(9)
N(5)–Hg(3)–O(18)	92.83(9)	O(18)–Hg(3)–O(22)	139.98(8)
N(5)–Hg(3)–O(17)	92.27(9)	O(21)–Hg(3)–O(22)	51.70(9)
N(5)–Hg(3)–O(21)	91.10(10)		
Hg(4)			
N(7)–Hg(4)–O(25)	120.80(10)	N(8)–Hg(4)–O(30)	104.80(9)
N(7)–Hg(4)–O(26)	82.95(9)	O(25)–Hg(4)–O(26)	52.24(9)
N(7)–Hg(4)–O(29)	133.5(10)	O(25)–Hg(4)–O(29)	96.41(9)
N(7)–Hg(4)–O(30)	82.74(9)	O(25)–Hg(4)–O(30)	136.00(9)
N(7)–Hg(4)–N(8)	104.2(10)	O(26)–Hg(4)–O(29)	143.39(9)
N(8)–Hg(4)–O(26)	80.87(9)	O(26)–Hg(4)–O(30)	165.52(8)
N(8)–Hg(4)–O(25)	104.10(10)	O(29)–Hg(4)–O(30)	50.76(9)
N(8)–Hg(4)–O(29)	91.2(10)		

**Figure 4:**
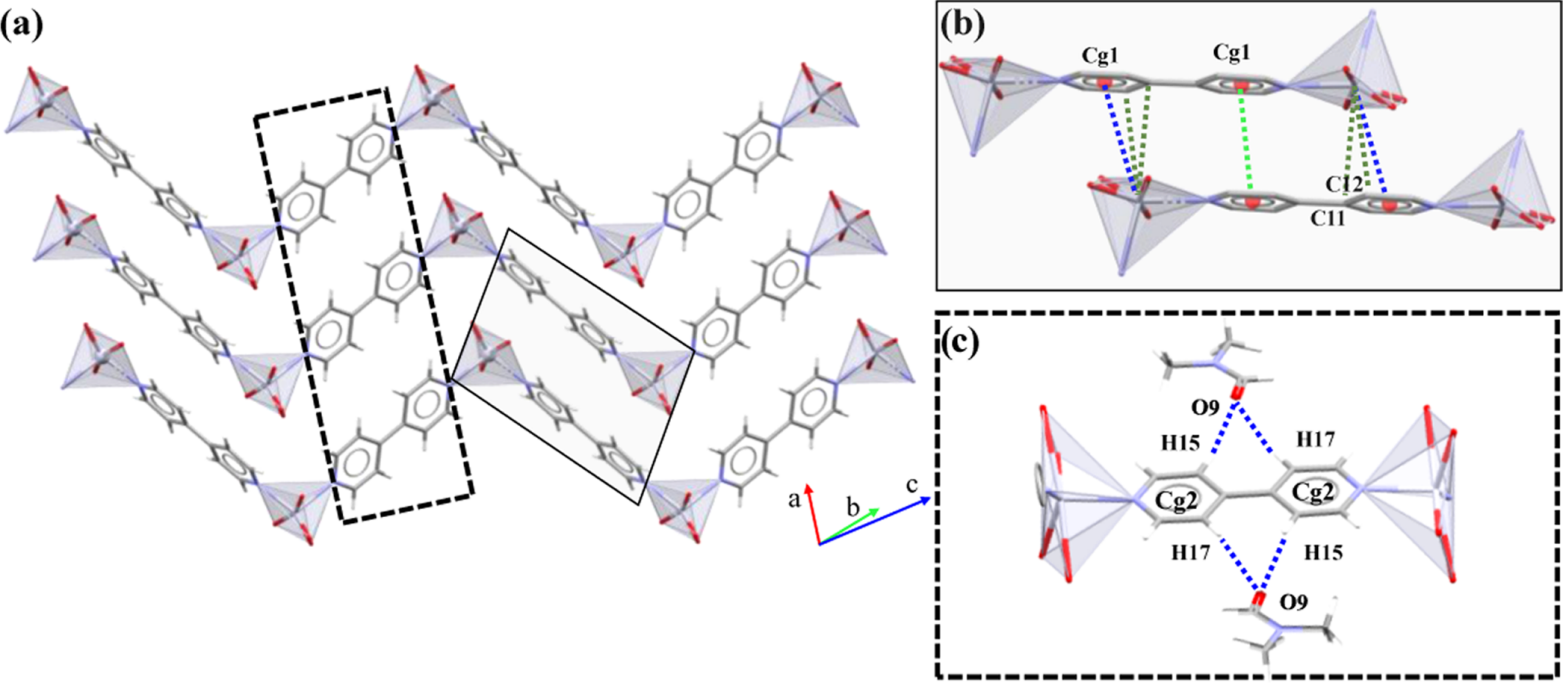
(a) Stacking of chains in **P1A** through (b) π···π and Hg···π interactions or (c) between DMF and 4,4′-bipy by C–H···O interactions. Hg···C interactions are highlighted in dark green and correspond to Hg1···C12, 3.52(1) Å, and Hg1···C11, 3.801(9) Å. Hg···C contact: Hg1···C12, 3.52(1) Å; Hg1···C11, 3.801(9) Å. Cg(2) = N2–C14–C15–C16–C17–C18.

**P1A** has one alternated cooperative interaction (Figure 4a), in which planar aromatic rings of 4,4′-bipy stacks at 3.736 Å together with a Hg···π interaction at 3.953 Å (Figure 4b). The occluded DMF molecules are responsible for the coplanar orientation of 4,4′-bipy. They act as double C–H···O acceptors by associating with 4,4′-bipy through its four *m*-H atoms and fixing the two pyridyl rings (Table 2). As a consequence, the 4,4′-bipy ligands are coplanar preventing the formation of π···π interactions (Figure 4c).

**Figure 5:**
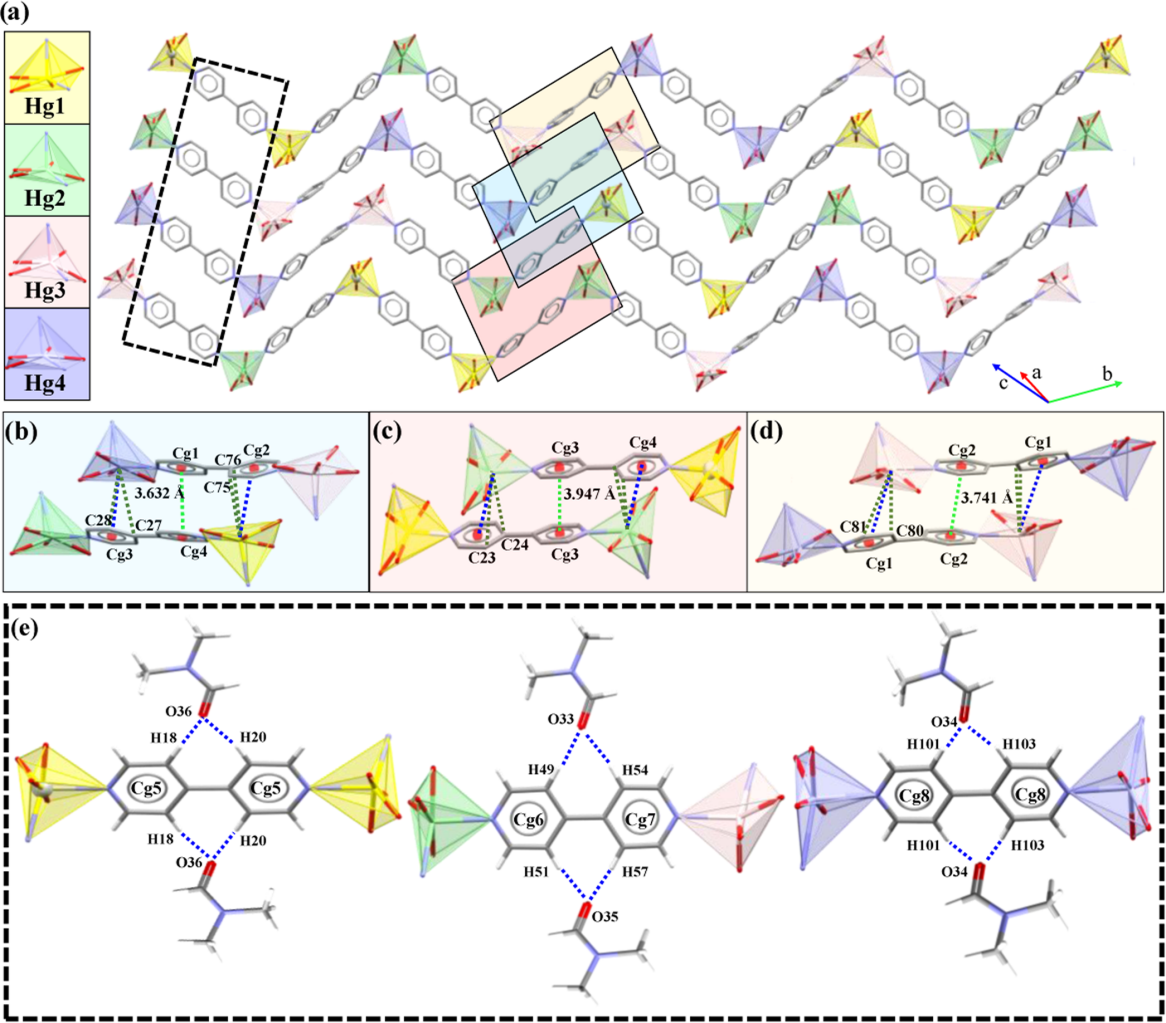
Stacking of chains in **P1B**. Inset of π···π and Hg···π interactions between (a) Hg3 and Hg4; (b) between Hg1 and Hg4; (c) Hg1 and Hg2, and (d) Hg3 and Hg4. (e) C–H···O interactions between pyridyl rings of 4,4′-bipy and DMF. Color codes: Hg1 in yellow, Hg2 in light green, Hg3 in pink, and Hg4 in light violet. Blue dashed lines indicate Hg···π interactions in (b)–(d) and C–H···O in (e). π···π interactions are shown as light green dashed lines. Hg···C contacts: Hg1···C75, 3.641(3) Å; Hg1···C76, 3.769(3) Å; Hg2···C23, 3.628(3) Å; Hg2···C24, 3.867(3) Å; Hg3···C80, 3.396(3); Hg3···C81, 3.816(3); Hg4···C28, 3.315(4) Å; Hg4···C27, 3.737(3) Å. Cg(5) = N1–C17–C18–C19–C20–C21; Cg(6) = N4–C48–C49–C50–C51–C52; Cg(7) = N5–C53–C54–C55–C56–C57; Cg(8) = N8–C100–C101–C102–C103–C104.

**P1B** emulates the alternated sequence of interactions present in **P1A** but splitting each of the two patterns into three (Figure 5a).

It exhibits cooperative π···π and Hg···π interactions involving Hg1–Hg4 (Figure 5b), Hg1 and Hg2 (Figure 5c), or Hg3 and Hg4 (Figure 5d) and three different C–H··O interactions with the occluded DMF molecules (Figure 5e and Table 5). The Hg–C_π_ contact value of 3.52(1) Å in **P1A** and between 3.315(4) and 3.641(3) Å in **P1B** fall within the range of previously reported examples.^67^

**Table 5:** Intermolecular Interactions Present in **P1B**^a^

**P1B**	H···A (Å)	D···A (Å)	D–H (Å)	>D–H···A (deg)
C(49)–H(49)···O(33)	2.291	3.203(5)	0.950	160.8
C(54)–H(54)···O(33)	2.438	3.339(4)	0.950	158.3
C(51)–H(51)···O(35)	2.333	3.245(5)	0.950	160.8
C(57)–H(57)···O(35)	2.396	3.281(6)	0.950	154.9
C(101)–H(101)···O(34)	2.310	3.225(6)	0.950	161.6
C(103)–H(103)···O(34)	2.434	3.352(5)	0.950	162.4
C(18)–H(18)···O(36)	2.312	3.222(5)	0.950	160.2
C(20)–H(20)···O(36)	2.495	3.413(4)	0.950	162.5

π interactions			
Cg(*I*)···Hg(*J*)	Cg···Hg^b^	MeJ_Perp	β
Cg(1)···Hg(3)	4.004		
Cg(2)···Hg(1)	3.850	3.619	19.93
Cg(3)···Hg(4)	3.867	3.228	33.42
Cg(4)···Hg(2)	4.121		

Cg(*I*)···Cg(*J*)	Cg···Cg^c^	α^d^	β, γ^e^	Cg(*I*)_Perp, Cg(*J*)_Perp^f^	slippage^g^
Cg(1)···Cg(4)	3.632(2)	6.1(2)	24.7, 18.6	3.442(1), 3.301(1)	1.516
Cg(2)···Cg(2)	3.741(5)	0.03(2)	5.0, 5.0	3.727(1), 3.727(1)	0.323
Cg(3)···Cg(3)	3.947(2)	0.02(16)	33.1, 33.1	3.306(1), 3.306(1)	2.156

a Cg···Cg and Cg···Hg distances are given in Å.

b Cg···Hg = distance between ring centroid and Hg(II) center.

c Cg···Cg = distance between ring centroids (Å).

d α = dihedral angle between planes I and J (deg).

e Offset angles: β = angle Cg(*I*)–Cg(*J*) and normal to plane *I* (deg) and γ = angle Cg(*I*)–Cg(*J*) and normal to plane *J* (deg) (β = γ, when α = 0).

f Perpendicular distance (Å) of Cg(*I*) on plane *J* and perpendicular distance (Å) of Cg(*J*) on plane *I* (equal when α = 0).

g Slippage = horizontal displacement or slippage between Cg(*I*) and Cg(*J*) (equal for both centroids when α = 0). Cg(1) = N2–C22–C23–C24–C25–C26; Cg(2) = N6–C74–C75C76–C77–C78; Cg(3) = N3–C27–C28–C29–C30–C31; Cg(4) = N7–C79–C80–C81–C82–C83.

**Figure 6:**
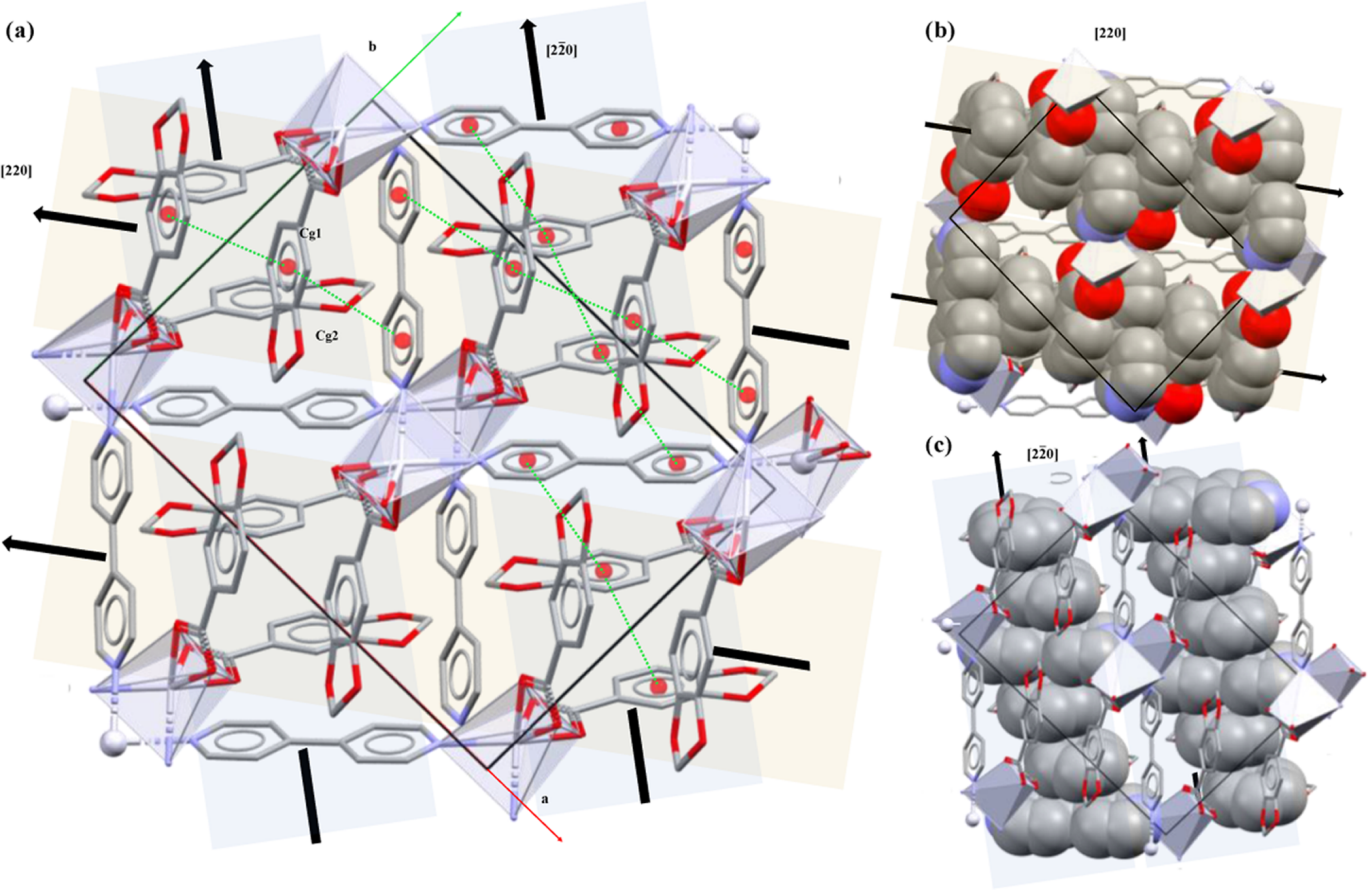
(a) Crystal packing of **2** assembled by π···π interactions along [220] (highlighted in light orange) or [22̅0] direction (highlighted in light blue). Spacefill representation of the aromatic rings stacked along (b) [220] or (c) [22̅0]. Hydrogen atoms are omitted for clarity.

By the same token, **2** assembles through π···π stacking but in a different manner. The piling of the chains is set in the alternate sequence −Pip–Pip–4,4′-bipy– from perpendicular polymeric chains (Figure 6a). The aromatic rings of the Pip ligands stack at a Cg(1)···Cg(1) distance of 3.906(2) Å, whereas the interaction between Pip and 4,4′-bipy is of Cg(1)···Cg(2), 4.000(2) Å (Table 6). This sequence is repeated twice within the unit cell along [220] (Figure 6b) or [22̅0] (Figure 6c) directions.

**Table 6:** Bond Lengths (Å), Bond and Torsion Angles (deg), and Intermolecular Interactions Present in **2**^a^

bond lengths			
Hg(1)–O(1)#1	2.1896(19)	Hg(1)–N(1)#1	2.282(2)
Hg(1)–O(2)	2.993(2)		

bond angles			
O(1)–Hg(1)–N(1)	132.82(7)	O(1)–Hg(1)–O(1)	99.41(12)
O(1)#1–Hg(1)–N(1)	103.67(8)	O(1)–Hg(1)–O(2)	48.24(12)
N(1)–Hg(1)–N(1)#1	89.82(10)	O(1)–Hg(1)–O(2)#1	98.90(12)
N(1)–Hg(1)–O(2)	127.06(7)	O(2)–Hg(1)–O(2)#1	133.42(10)
N(1)–Hg(1)–O(2)#1	87.56(8)		

Cg(*I*)–Cg(*J*)	torsion angle, χ
Cg(1)–Cg(1)	0.1(3)

π interactions					
Cg(*I*)···Cg(*J*)	Cg···Cg^b^	α^c^	β, γ^d^	Cg(*I*)_Perp, Cg(*J*)_Perp^e^	slippage^f^
Cg(1)···Cg(1)	3.906(2)	0	21.3, 21.3	3.640(1), 3.640(1)	1.418
Cg(1)···Cg(2)	4.000(2)	1.83(12)	30.2, 31.0	3.427(1), 3.457(1)	

a #1 −*x* + 1, *y*, −*z* + 1/2. Cg···Cg distances are given in Å. Cg···Cg and Cg···Hg distances are given in Å.

b Cg···Cg = distance between ring centroids (Å).

c α = dihedral angle between planes I and J (deg).

d Offset angles: β = angle Cg(*I*)–Cg(*J*) and normal to plane *I* (deg) and γ = angle Cg(*I*)–Cg(*J*) and normal to plane *J* (deg) (β = γ, when α = 0).

e Perpendicular distance (Å) of Cg(*I*) on plane *J* and perpendicular distance (Å) of Cg(*J*) on plane *I* (equal when α = 0).

f Slippage = horizontal displacement or slippage between Cg(*I*) and Cg(*J*) (equal for both centroids when α = 0). Cg(1) = C2–C3–C4–C6–C7–C8; Cg(2) = N1–C9–C10–C11–C12–C13.

These π···π interactions are supported by C–H···O interactions, but, unlike **P1A** and **P1B**, only two *m*-H of 4,4′-bipy pointing in opposite directions act as donors and interact with two coordinated carboxylate O atoms from parallel chains or with two dioxole O atoms from perpendicular chains.

### Hirshfeld Surfaces and 2D Fingerprint Plots of **P1A**, **P1B**, and **2**

Intermolecular interactions have been analyzed using CrystalExplorer 17.5^41^ by Hirshfeld Surface and 2D fingerprint plot analyses. The C–H···O interactions are highlighted as red spots in the lateral region of the Hirshfeld surfaces surrounding 4,4′-bipy ligands (Figure S16, Supporting Information). They are displayed in the 2D fingerprint plot as broad wings representing a total of 27.3% (**P1A**), between 27.0 and 28.0% (**P1B**) or 28.0% (**2**) of contact surface. The complementary interactions from the DMF molecules are found in the Supporting Information (Figure S17). Besides, π···π interactions are pointed as a central region in the 2D fingerprint plot that corresponds to the 4.1% (**P1A**), between 2.7 and 4.1% (**P1B**) or to a 14.1% (**2**) of C–C contact surface. It should be mentioned that despite the marked contribution from planar interactions in **2**, these aromatic rings are on the brink of effective π···π interactions. Finally, Hg(II) centers display a 3.4% (**P1A**) or between 3.0 and 3.4% (**P1B**) of contact surface toward the aromatic rings of 4,4′-bipy, while no contribution is observed in **2** (Table S4, Supporting Information).

### DFT Calculations

With the aim of getting further insights into the relative stabilities of **P1A** and **P1B** polymorphs, we performed periodic DFT(PBE-D2) calculations with VASP code.^44,45^ The DFT optimized structures are close to those determined by single-crystal X-ray diffraction and particularly both the distorted pentagonal Hg(II) geometry and the distance between 1D zigzag polymeric chains are essentially unchanged. This suggests that the present methodology accounts for the subtle van der Waals interactions. According to calculations, **P1B** is more stable than **P1A** by 13.4 kJ·mol^–1^ per [Hg(Pip)_2_(μ-4,4′-bipy)]·DMF formula unit. The energy difference between the two polymorphs is small, and this is in agreement with the formation of the two polymorphs and the possibility of selectively obtaining one of the two species. A partition energy scheme (Table 7) was performed to determine the origin for the preference for **P1B**. We decomposed the association energy (*E*) between two metal dimers of vicinal chains (see Figure S18 for the model systems used in the partition scheme) in two terms: (i) distortion of the two dimers with respect to a hypothetical isolated dimer and (ii) the interaction energy between the distorted fragments (further details can be found in the Experimental Section). Results indicate that the metal dimer distortion energies are all very similar (the largest variation is 4.9 kJ·mol^–1^). In contrast, the interaction energy between the distorted fragments differs significantly, and it is larger for the vicinal chains of **P1B**. Consequently, the association between vicinal chains is 21.0 and 36.1 kJ·mol^–1^ stronger for **P1B** than for **P1A**, and this suggests that the thermodynamic preference for **P1B** originates mainly from metal cation···π and π···π interactions, rather than the distortion arising from crystal packing. Remarkably, Hg(3) and Hg(4) centers, which present the less conventional coordination environment, show the smallest distortion energies and the highest interaction energies, thus suggesting that the final geometry around the metal center is tuned by several factors.

**Table 7:** Energy Partition Scheme Analysis of the Association Energy between Vicinal Chains Following Equation 1^a^

fragment	*E*	*E*_DIS(1)_	*E*_DIS(2)_	*E*_INT_
**P1A**				
Hg(1)–Hg(1)/Hg(1)–Hg(1)	–144.8	–76.1	–76.1	–296.9
**P1B**				
Hg(2)–Hg(1)/Hg(1)–Hg(2)	–170.1	76.9	76.9	–323.9
Hg(3)–Hg(4)/Hg(4)–Hg(3)	–180.9	72.0	72.0	–324.9
Hg(2)–Hg(1)/Hg(4)–Hg(3)	–165.8	76.9	72.0	–314.7

a *E* is the association energy, *E*_DIS(1)_ and *E*_DIS(2)_ are the energies required to distort the dimers to achieve the crystal structure, and *E*_INT_ is the interaction energy between distorted fragments. All values are given in kJ·mol^–1^.

### Photoluminescence Studies

**Figure 7:**
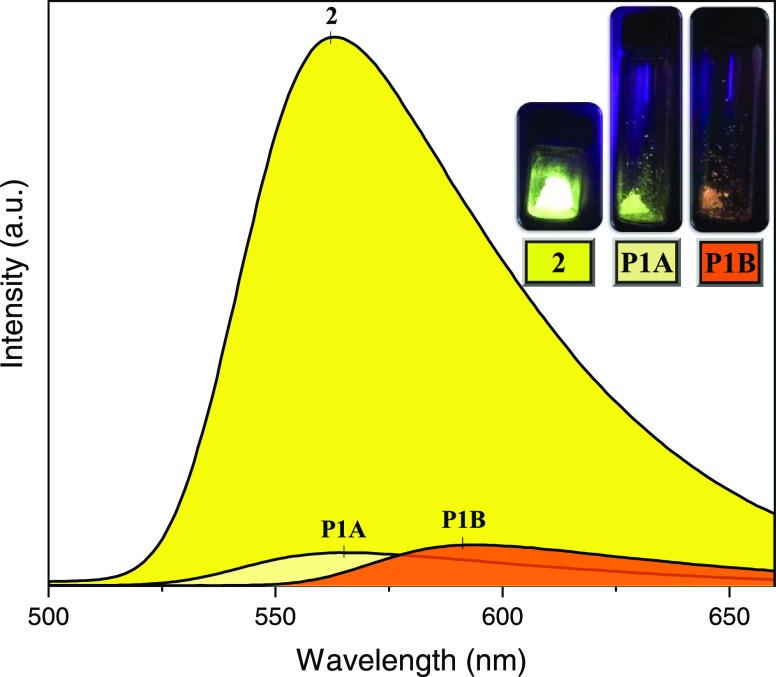
Solid-state emission spectra of complexes **2** (λ_max-em_ = 562 nm, bright gold), **P1A** (λ_max-em_ = 565 nm, mikado yellow), and **P1B** (λ_max-em_ = 591 nm, bright orange). Inset of the samples under UV light of λ = 335 nm exposure.

Photoluminescence properties were recorded using single crystals of **P1A**, **P1B**, and **2** (Figure S19, Supporting Information). Under UV excitation of a pulse laser beam at λ_exc_ = 335 nm, the samples display the corresponding emission spectra with emission maxima (λ_max-em_) located at 565 nm (**P1A**), 591 nm (**P1B**), and 562 nm (**2**). The three spectra are composed of unstructured bands suggesting charge transfer transitions in character (Figure 7). The formation of the coordination polymers caused a bathochromic shift in emission compared to the free 4,4′-bipy ligand (λ_max-em_ = 401 nm).^68^ The emission of **P1B** is considerably blue-shifted, whereas **P1A** and **2** present closer emission maxima. All of them present moderate Stokes shifts of 12 152 cm^–1^ (**P1A**), 12 930 cm^–1^ (**P1B**), and 12 057 cm^–1^ (**2**). As displayed in CIE 1931 chromaticity diagrams, both **P1A** and **2** display yellow emission colors (λ_max-em_), while **P1B** is reddish-orange at being irradiated under the selected λ_exc_ (Figure S20, Supporting Information). A thorough study of the photophysical properties of biphenyl and related aromatic ligands^69^ evinced how molecular structure affects the shape and wavelength position of the emission spectrum. The case of biphenyl molecule remarks how the gain or loss of planarity modifies the absorption and emission properties. Since its increase is rendered to sharper spectra and better quantum yields, the loss of planarity tends to provoke larger stokes shifts. Noteworthily strong π···π stacking as well as Hg···π interactions open relaxation pathways to nonradiative decay processes and quench fluorescence.^6^ Therefore, the remarkable quenching in the emission of **P1A** and **P1B** compared to **2** could be explained by both the combination of intermolecular Hg···π and π···π interactions, being the former only present in **P1A** and **P1B**. Besides, the aromatic rings in **2** are on the brink of 4.0 Å for an effective π···π interaction (Table 6), whereas **P1A** and **P1B** have stacking of 4,4′-bipy at closer distances up to 3.736 (**P1A**) or 3.632 Å (**P1B**). By the same token, the larger stokes shift of **P1B** could be understood by considering the 4,4′-bipy torsion angles. Complexes **P1A** (χ = 1.8(11)°) and **2** (χ = 0.1(3)°) (Tables 2 and 6) present an adjacent λ_max-em_ instead, **P1B** has χ between 0.1(6) and 34.4(5)° (Table 3) with the consequent shift of emission up to 591 nm.

## Conclusions

We have successfully isolated two polymorphic forms (**P1A** and **P1B**), initially found to be concomitantly formed, as well as their desolvated form **2**. Interestingly, the separation between **P1A** and **P1B** was achieved by temperature or anion-template-dependent formation. Such control in polymorphism is scarce, especially when both forms tend to concomitantly crystallize. The crystal structures of the three compounds have been deeply analyzed revealing that Hg(II) is able to accommodate severe distortions and access to an uncommon distorted pentagonal pyramidal geometry. Those differences combined with the conformations of the 4,4′-bipy ligands resulted in significant variations of their photophysical properties. Besides, distortions in **P1B** not only modify the emission maxima, but, according to periodic-DFT calculations, they are counterbalanced leading to a more stable form as a consequence of stronger Hg(II)···π and π···π interactions. Therefore, this work contributes to the understanding of structure–property relationship in coordination polymers and provides an example of controlling the formation of concomitant polymorphs.

## Supporting Information Available

Further details of furnace conditions, PXRD patterns, FTIR-ATR, ^1^H NMR, ^13^C{^1^H} NMR, DEPT-135 NMR, and TG–DTA spectra as well as relevant data from Hirshfeld surfaces and 2D fingerprint plot analysis, *S* values, CIE 1931 chromaticity diagram and periodic DFT calculations (PDF)

## Accession Codes

CCDC 2101056–2101058 contain the supplementary crystallographic data for this paper. These data can be obtained free of charge via www.ccdc.cam.ac.uk/data_request/cif, or by emailing data_request@ccdc.cam.ac.uk, or by contacting The Cambridge Crystallographic Data Centre, 12 Union Road, Cambridge CB2 1EZ, UK; fax: +44 1223 336033.
