# Supplementary material for: Controlling the Formation of Two Concomitant Polymorphs in Hg(II) Coordination Polymers
Source: Inorg Chem. 2022 Mar 17;61(12):4965–79. doi: 10.1021/acs.inorgchem.1c03762 (PMC8965880; doi:10.1021/acs.inorgchem.1c03762)
Supplement: Supplementary file 1 — ic1c03762_si_001.pdf [file ic1c03762_si_001.pdf]

Supporting Information

Controlling the formation of two concomitant polymorphs in Hg(II) coordination polymers

Francisco Sánchez-Férez [a], *Xavier Solans-Monfort*[a], *Teresa Calvet*[b], *Mercè Font-Bardia*[c], *Josefina Pons*[a,*].

[a]Departament de Química, Universitat Autònoma de Barcelona, 08193-Bellaterra, Barcelona, Spain

[b]Departament de Mineralogia, Petrologia i Geologia Aplicada, Universitat de Barcelona, Martí i Franquès s/n, 08028 Barcelona, Spain.

[c]Unitat de Difracció de Raig-X, Centres Científics i Tecnològics de la Universitat de Barcelona (CCiTUB), Universitat de Barcelona, Solé i Sabarís, 1-3, 08028 Barcelona, Spain.

*Corresponding Author's E-mail: Josefina.Pons@uab.es

Figures Caption

Figure S1. Cooling ramp used in the synthesis of isolated **P1A** and **P1B**. 3
Figure S2. SC-XRD (top) and PXRD (bottom) patterns of **2**. 3
Figure S3. SC-XRD (top) and PXRD (bottom) patterns of **P1B**. 4
Figure S4. FTIR-ATR spectrum of **P1A** and **P1B** crystals mixture. 4
Figure S5. FTIR-ATR spectrum of isolated **P1A** crystals. 5
Figure S6. FTIR-ATR spectrum of isolated **P1B** crystals. 5
Figure S7. FTIR-ATR spectrum of compound **2** synthesized in DMF as solvent. 6
Figure S8. FTIR-ATR spectrum of compound **2** synthesized in milliQ as solvent. 6
Figure S9. FTIR-ATR spectrum of compound **2** synthesized in MeOH as solvent. 7
Figure S10. ^{1}H NMR spectrum of **P1A** and **P1B** mixture recorded at 298K in DMSO-d_6. 7
Figure S11. ^{1}H NMR spectrum of compound **2** recorded at 298K in DMSO-d_6. 8
Figure S12. $^{13}C\{^{1}H\}$ NMR (top) and DEPT-135 NMR (bottom) spectra of compound **2** recorded at 298K in DMSO-d_6. 9
Figure S13. TG-DTA of **2**. 10
Figure S14. TG-DTA of **P1A**. 10
Figure S15. TG-DTA of **P1B** 11
Figure S16. Hirshfeld surfaces and 2D fingerprint plots of complexes **P1A**, **P1B** and **2**. 11
Figure S17. Hirshfeld surfaces and 2D fingerprint plots of DMF molecules in complexes **P1A** and **P1B**. 12
Figure S18. Model used in the energy partitioning scheme. It includes two dimers of vicinal chains in **P1B**. 12
Figure S19. Samples of single crystals of (a) **2**; (b) **P1A** and (c) **P1B** used for the photophysical measurements. 13
Figure S20. Representation of the emission color of **P1A**, **P1B** and **2** within the CIE 1931 chromaticity diagram. 13

Tables Caption

Table S1. **P1A-P1B** relative energies per [Hg(Pip)$_2$(4,4'-bipy)]·DMF unit formula in kJ mol^{-1} as function of the Grimme's dispersion correction term. A positive value indicates that **P1A** is more stable than **P1B**. 14
Table S2. Unit cell measurements of **P1A** and **P1B** crystals 14
Table S3. Geometry distortions analysis using *S* parameter calculated with SHAPE[1,2] 15
Table S4. Percentage (%) of Hirshfeld surface implied in each contact for compounds **P1A, P1B** and **2**. 15

Furnace conditions

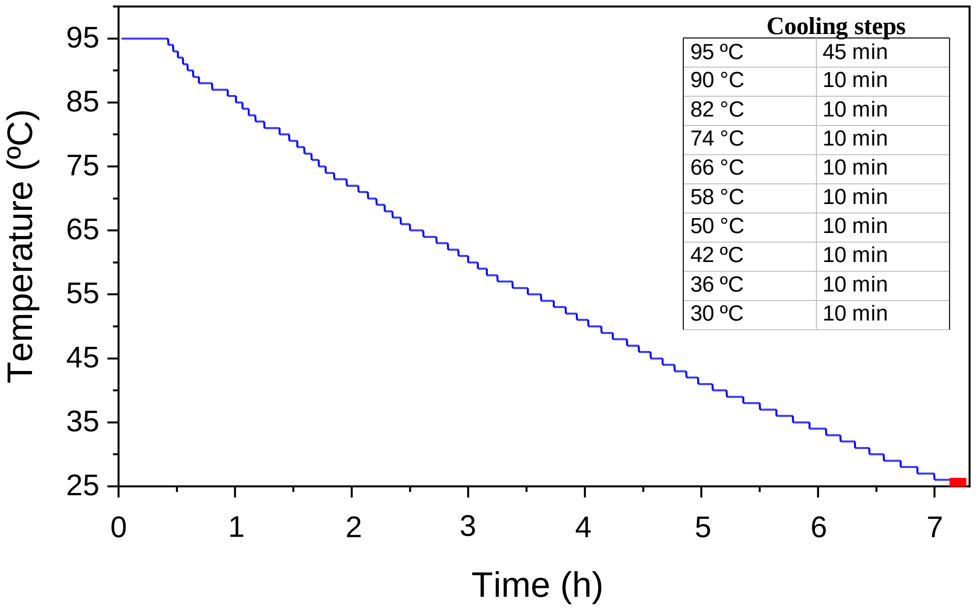

Figure S1. Cooling ramp used in the synthesis of isolated **P1A** and **P1B**.

PXRD patterns

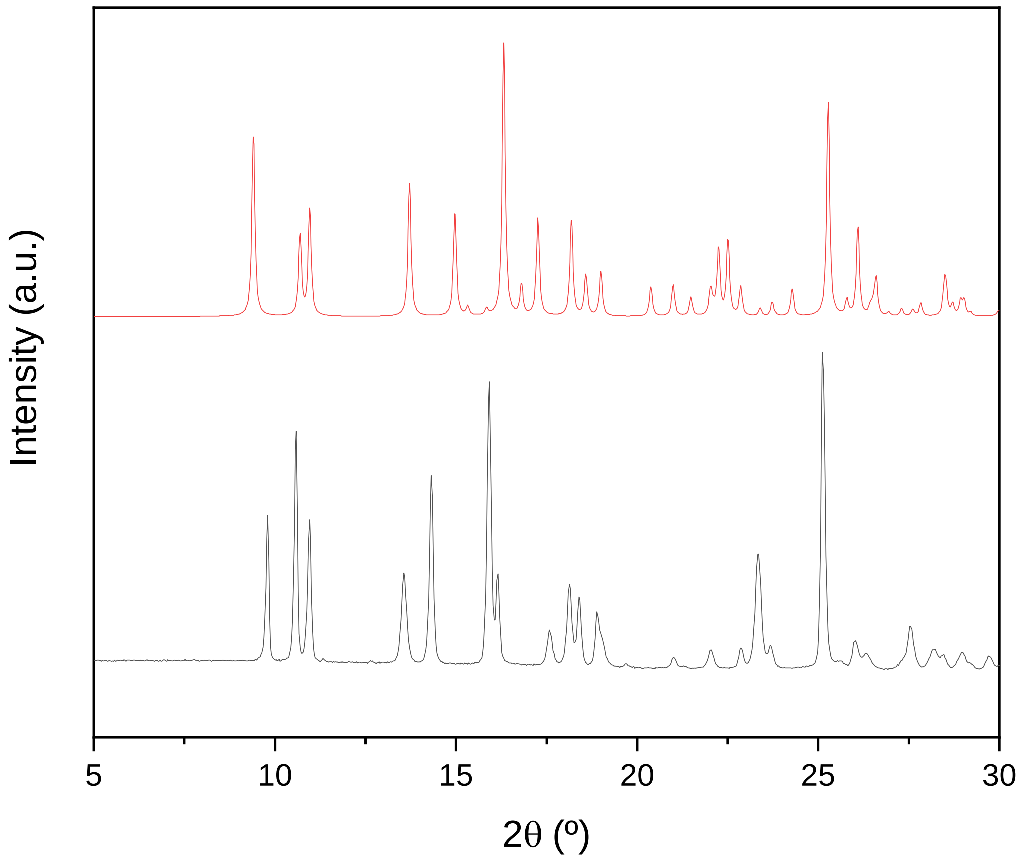

Figure S2. SC-XRD (top) and PXRD (bottom) patterns of **2**.

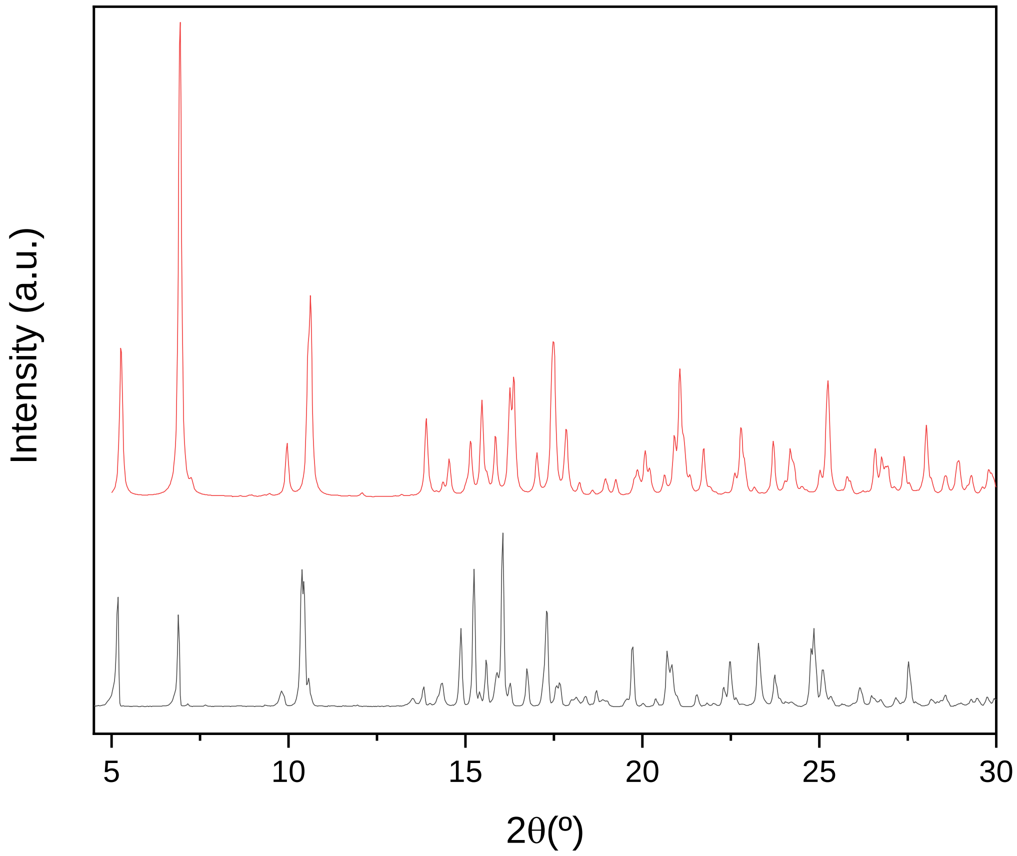

Figure S3. SC-XRD (top) and PXRD (bottom) patterns of **P1B**.

FTIR-ATR and ^{1}H, ^{13}C{^{1}H} and DEPT-135 NMR spectroscopies

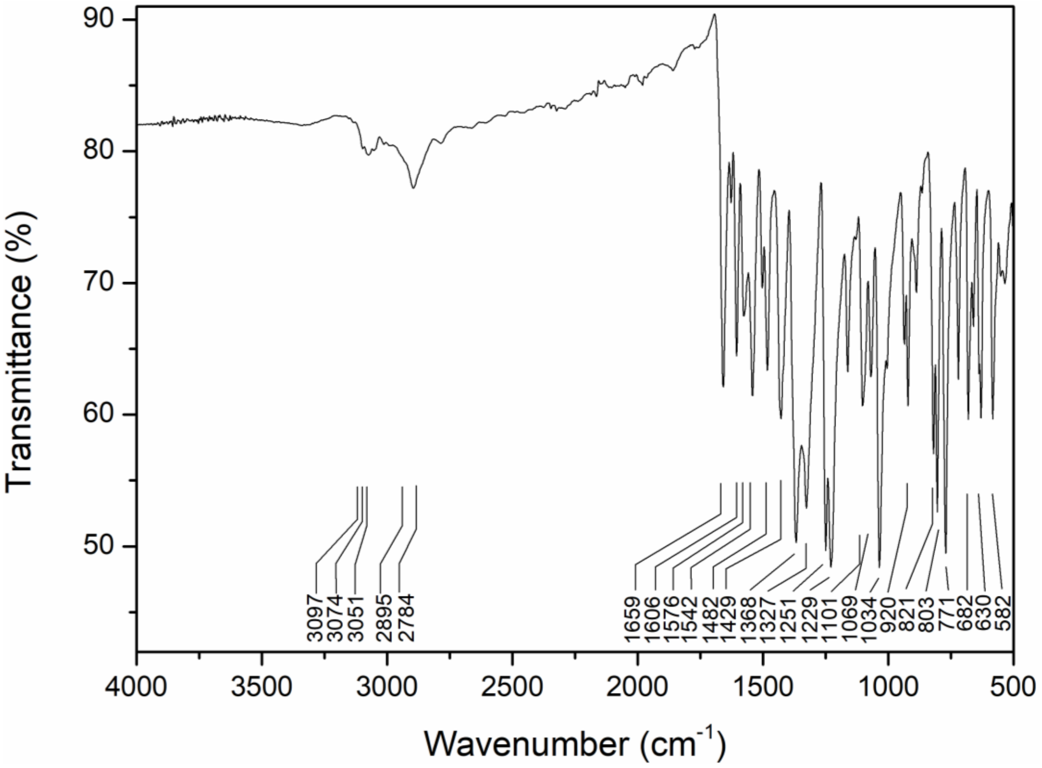

Figure S4. FTIR-ATR spectrum of **P1A** and **P1B** crystals mixture.

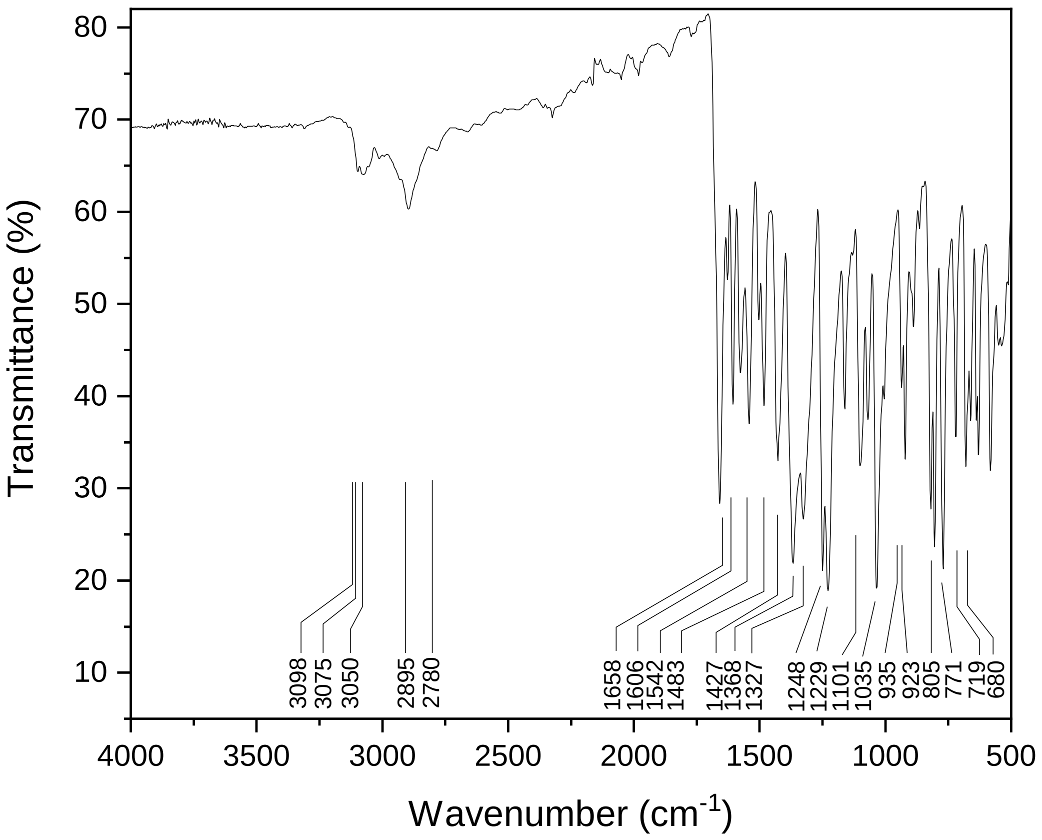

Figure S5. FTIR-ATR spectrum of isolated **P1A** crystals.

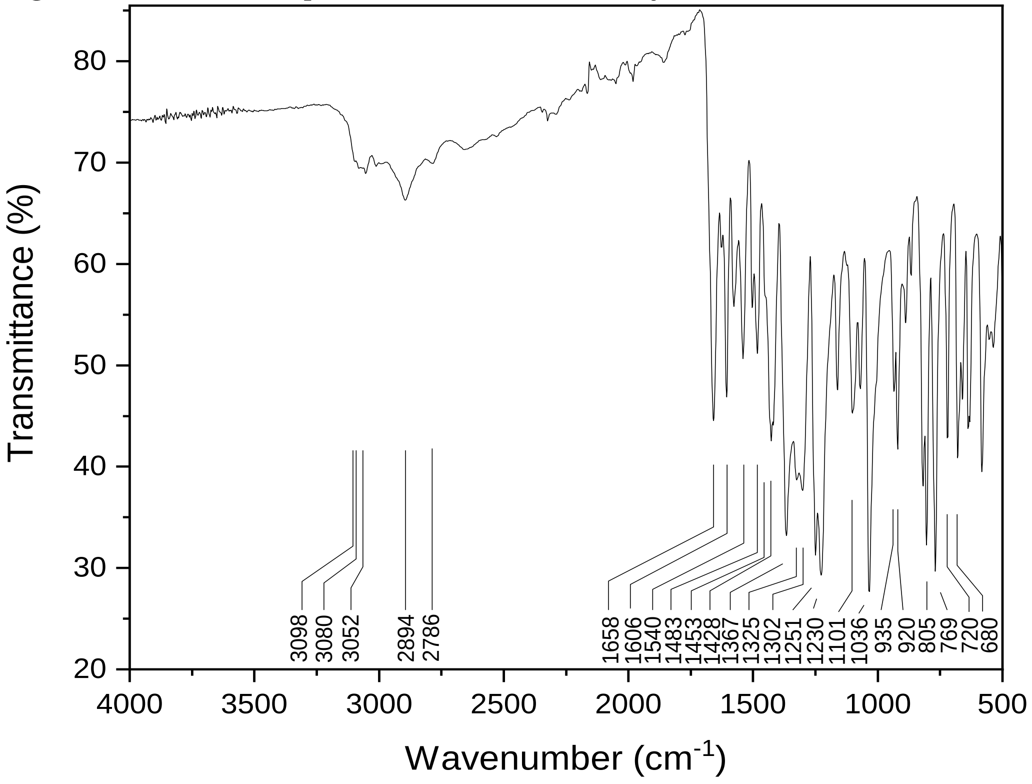

Figure S6. FTIR-ATR spectrum of isolated **P1B** crystals.

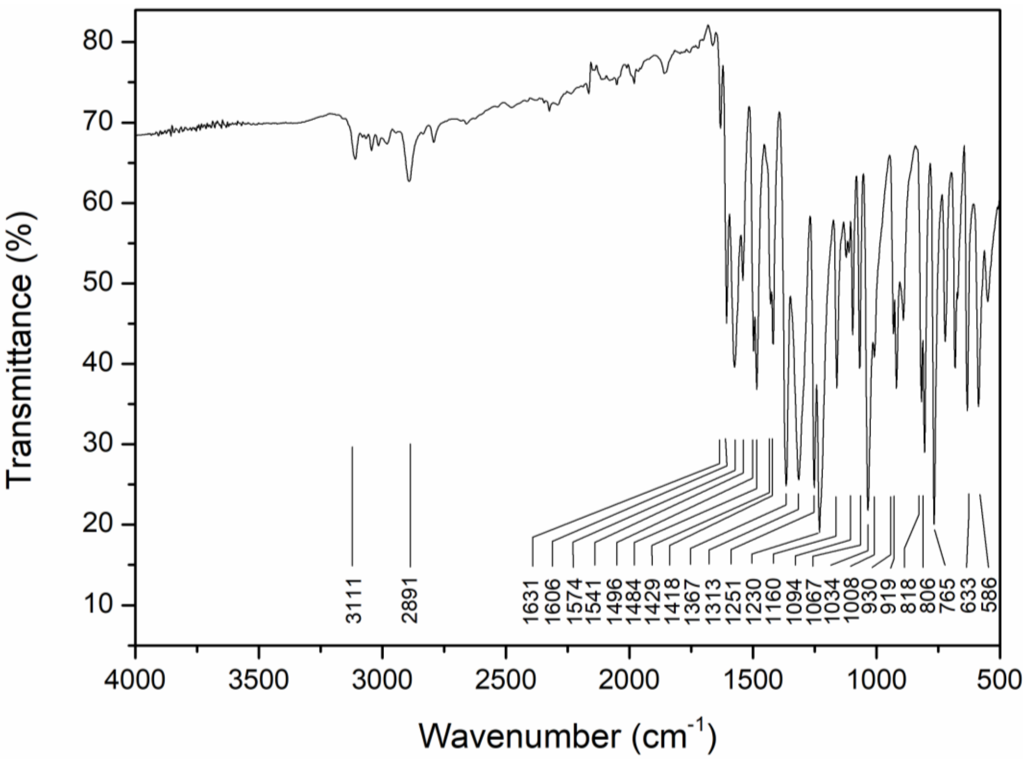

Figure S7. FTIR-ATR spectrum of compound **2** synthesized in DMF as solvent.

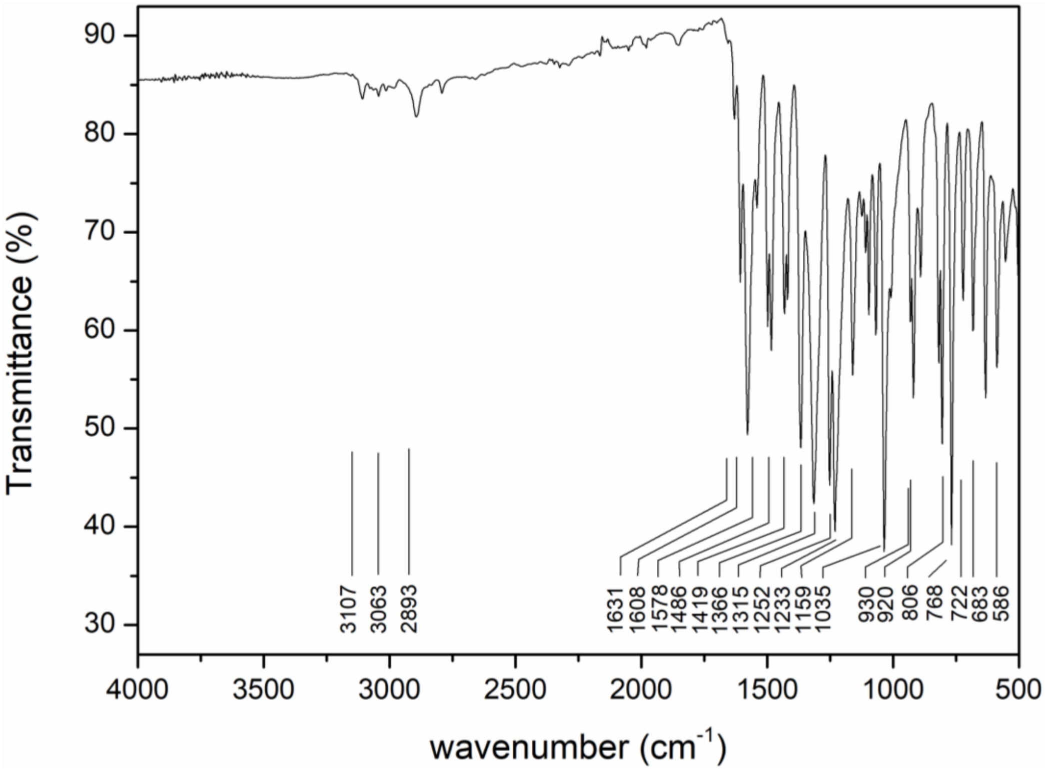

Figure S8. FTIR-ATR spectrum of compound **2** synthesized in milliQ as solvent.

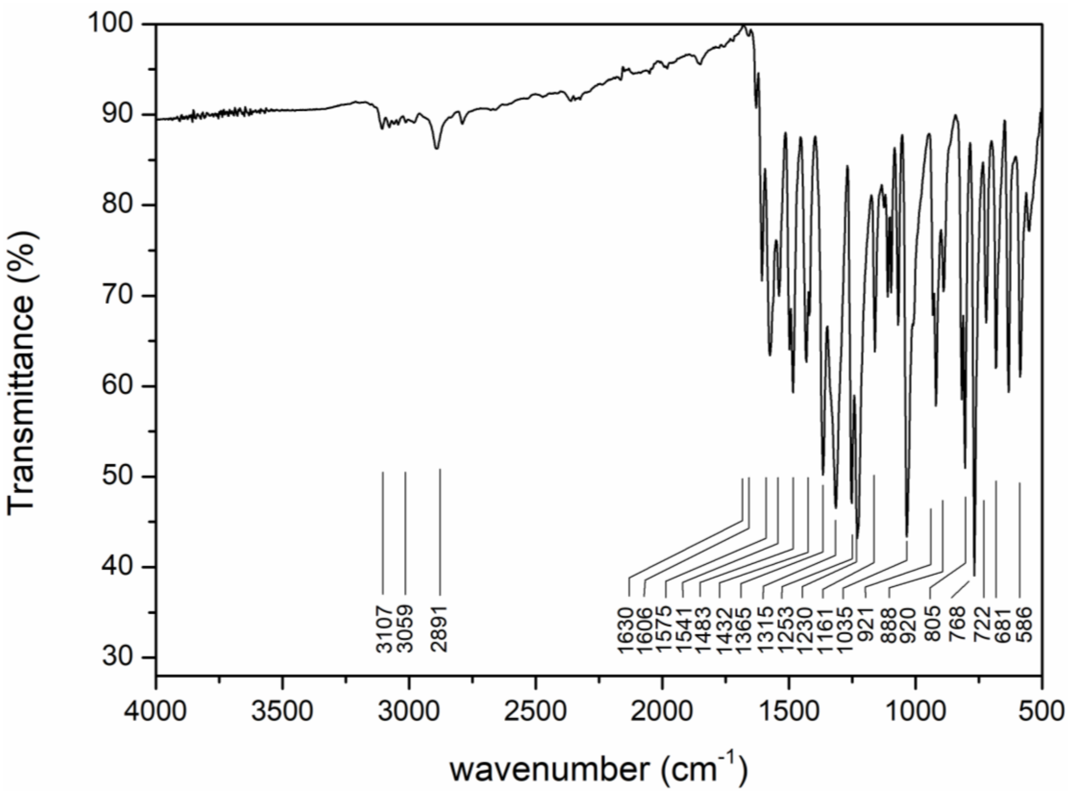

Figure S9. FTIR-ATR spectrum of compound **2** synthesized in MeOH as solvent.

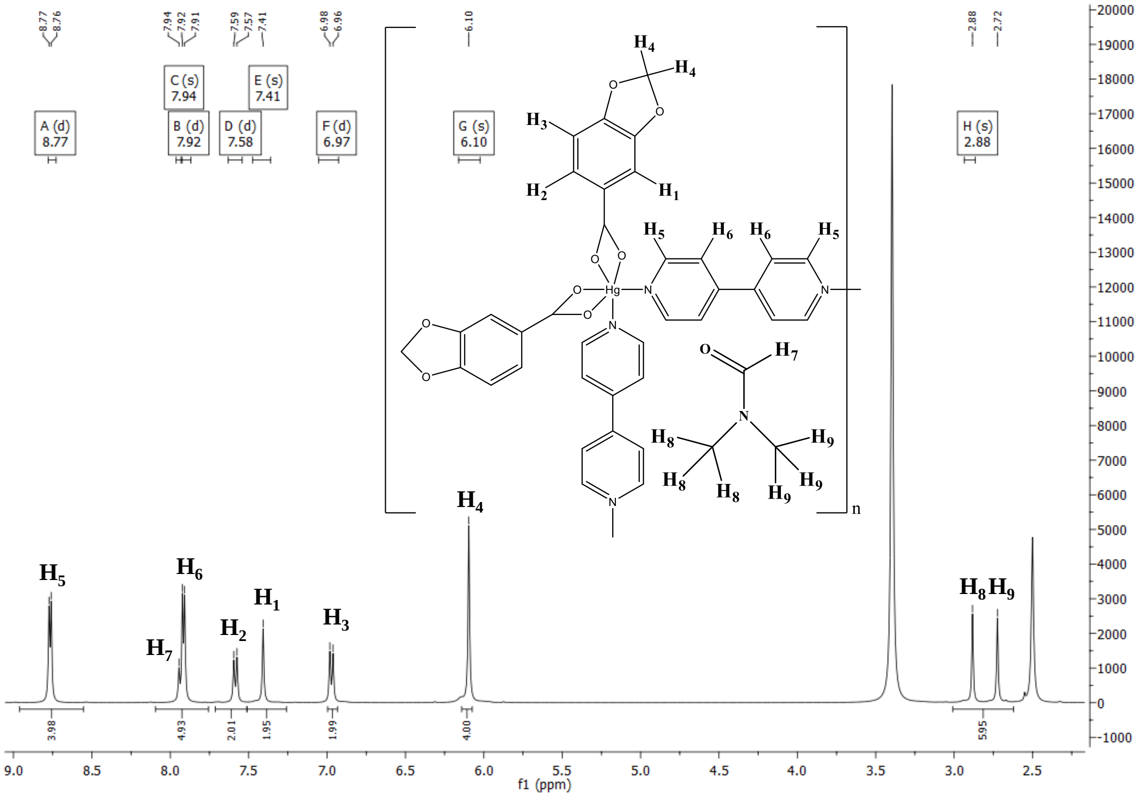

Figure S10. ^{1}H NMR spectrum of **P1A** and **P1B** mixture recorded at 298K in DMSO-*d*$_6$.

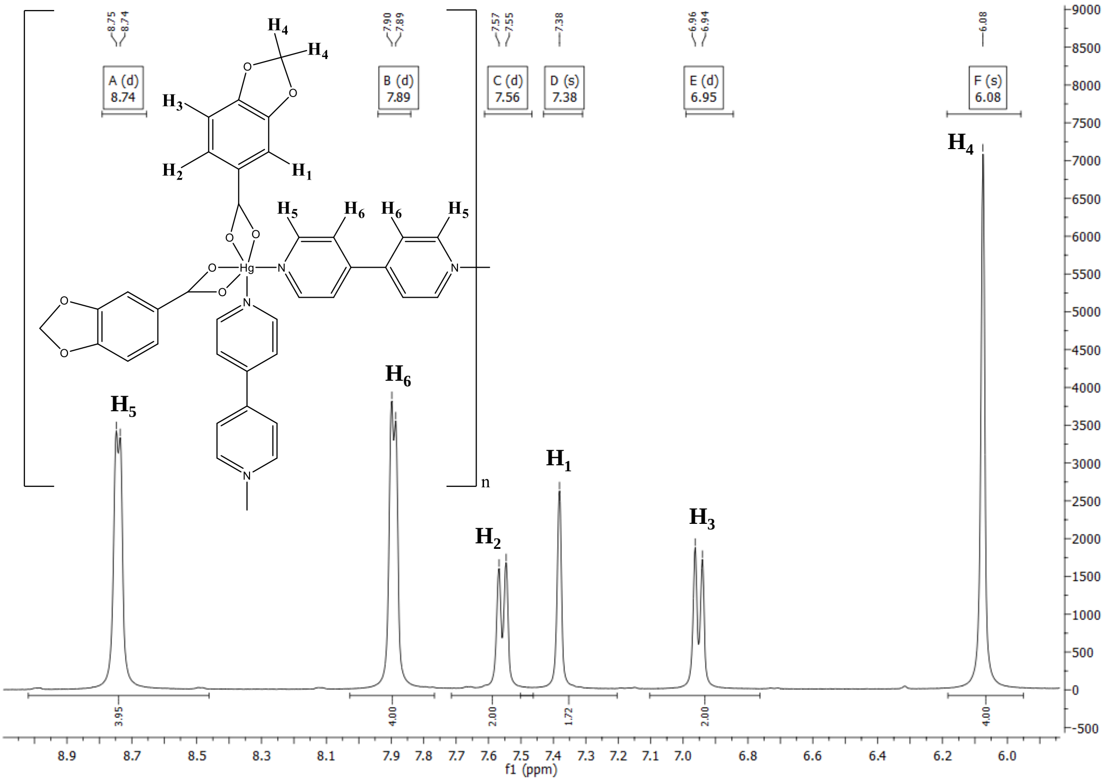

Figure S11. ^{1}H NMR spectrum of compound **2** recorded at 298K in DMSO-d_6.

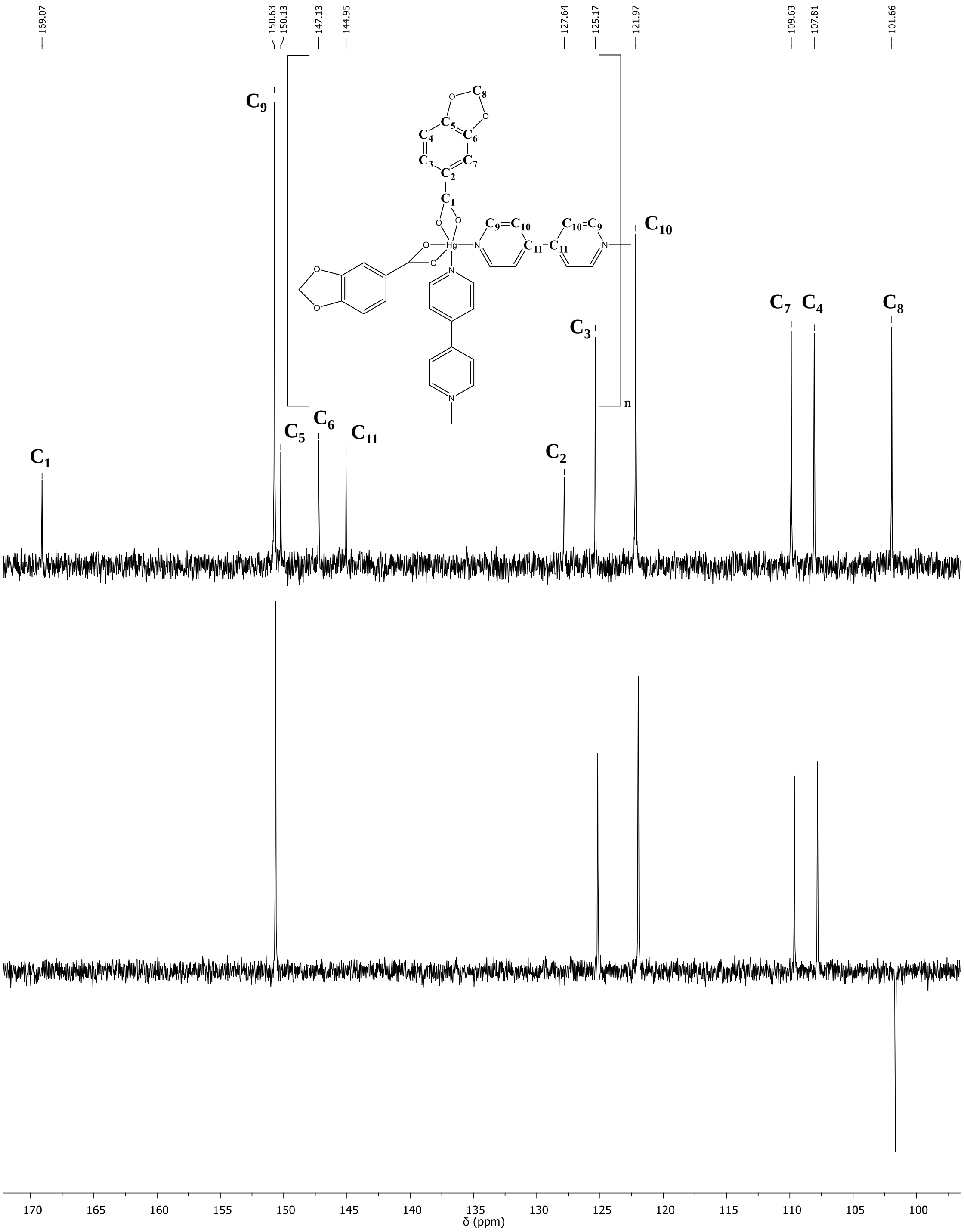

Figure S12. $^{13}C\{^{1}H\}$ NMR (top) and DEPT-135 NMR (bottom) spectra of compound **2** recorded at 298K in DMSO-d_6.

Thermogravimetric analysis

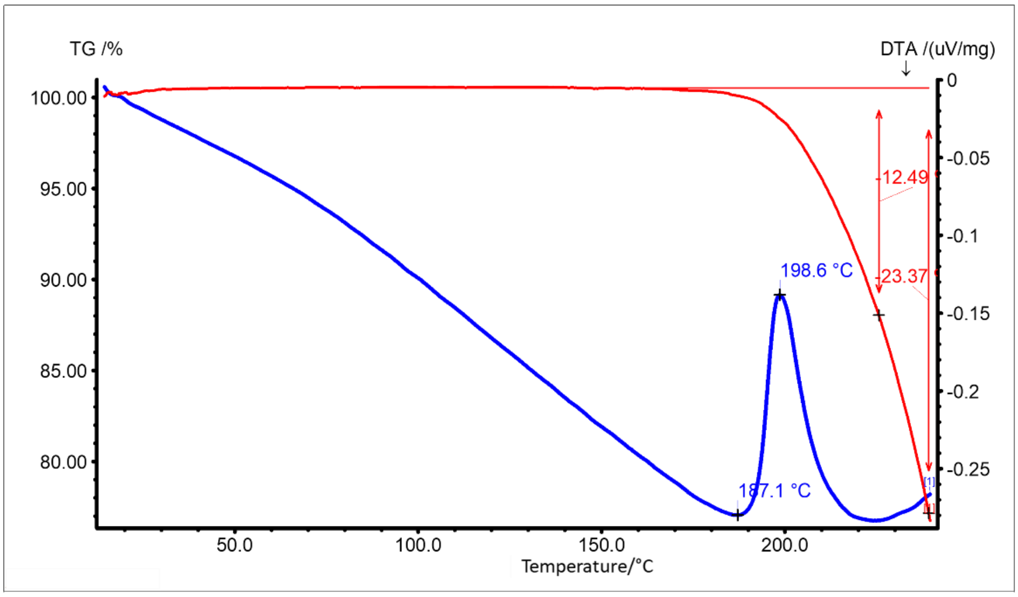

Figure S13. TG-DTA of **2**.

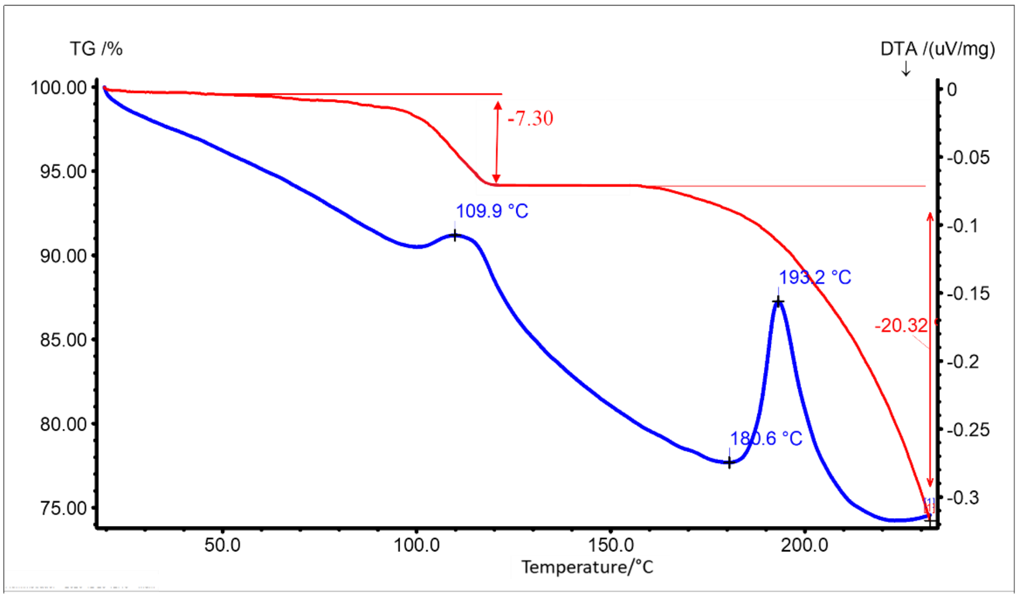

Figure S14. TG-DTA of **P1A**.

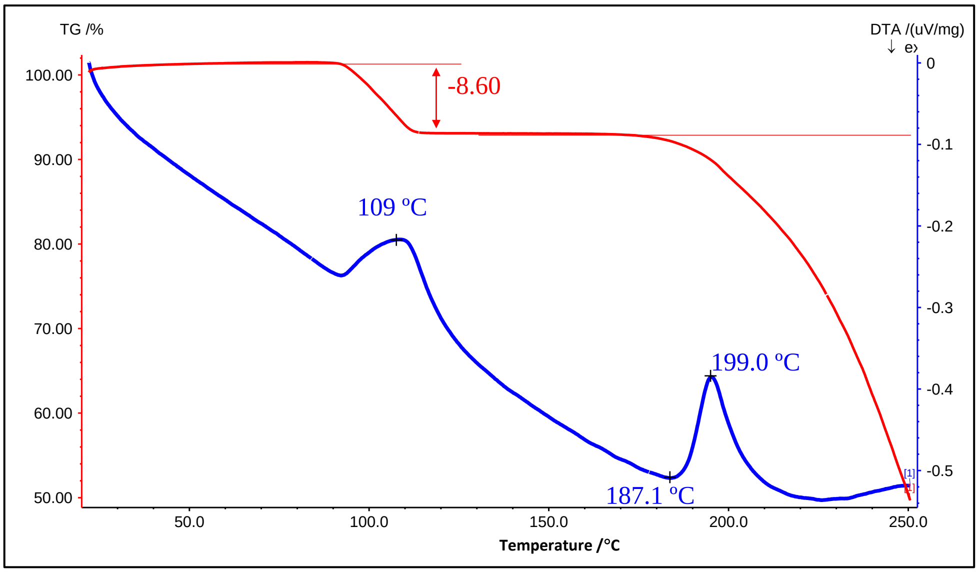

Figure S15. TG-DTA of **P1B**

Hirshfeld Surfaces and 2D fingerprint plot analyses

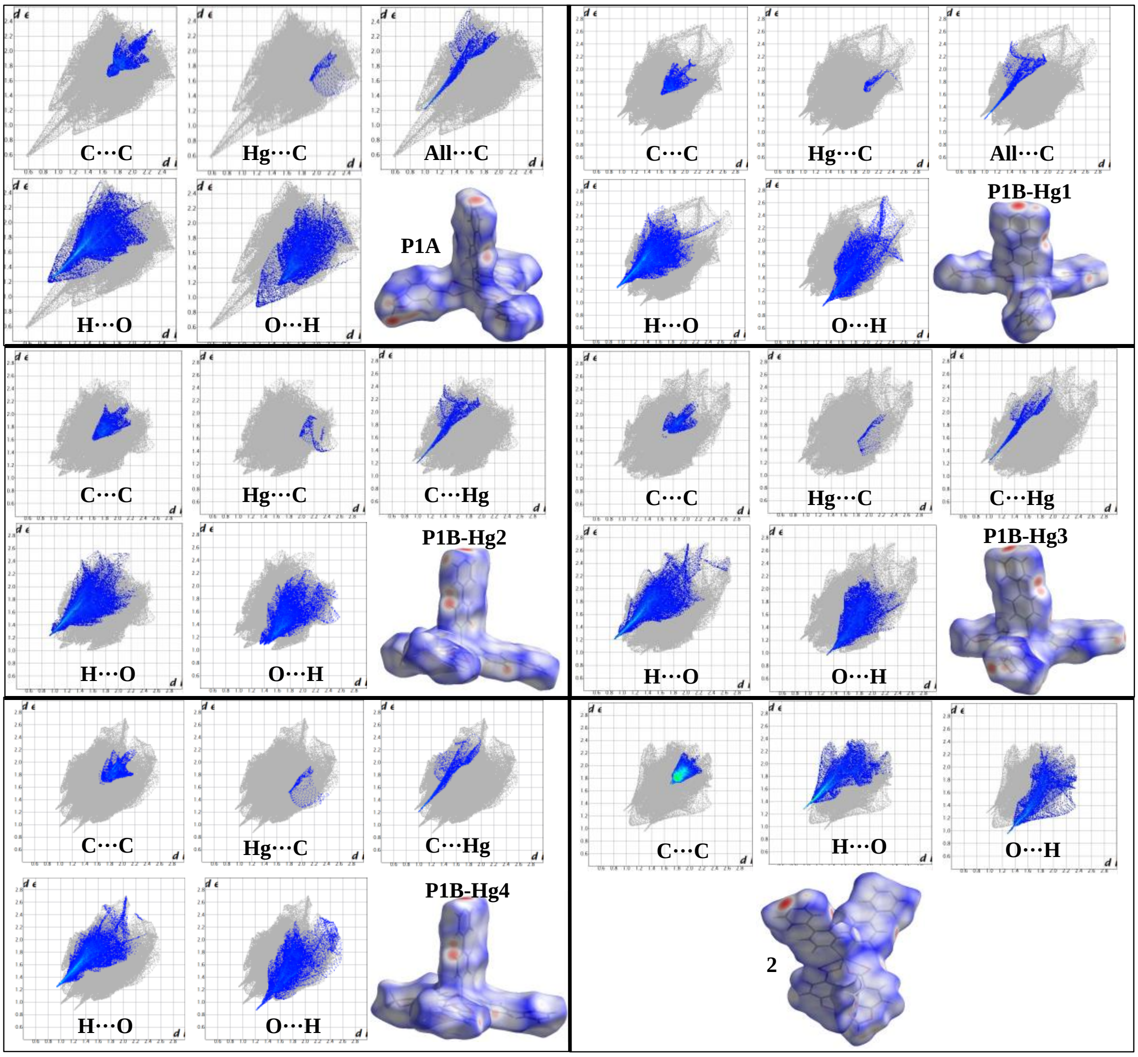

Figure S16. Hirshfeld surfaces and 2D fingerprint plots of complexes **P1A**, **P1B** and **2**.

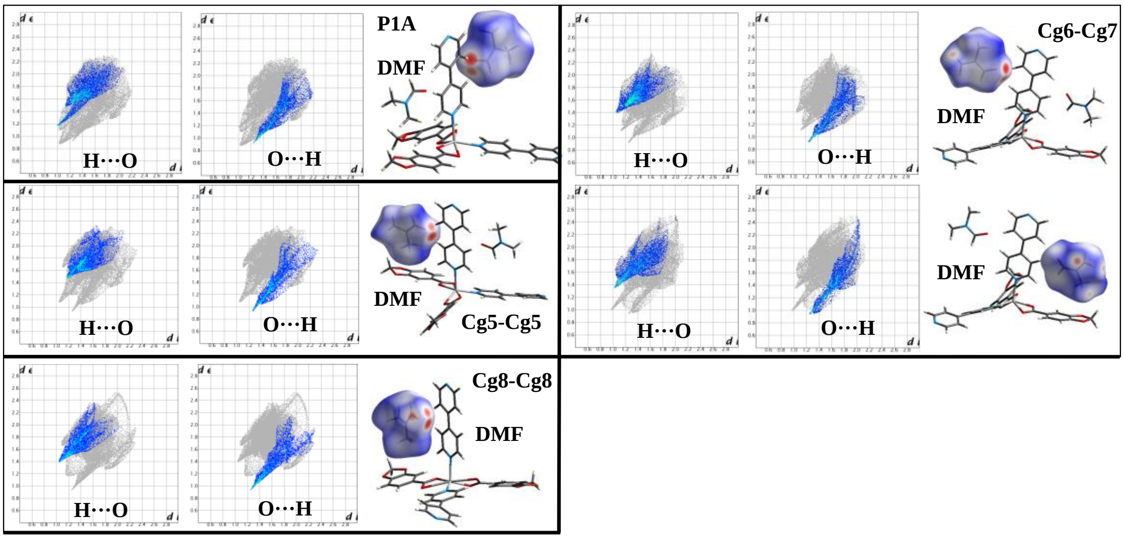

Figure S17. Hirshfeld surfaces and 2D fingerprint plots of DMF molecules in complexes **P1A** and **P1B**.

Energy partitioning scheme

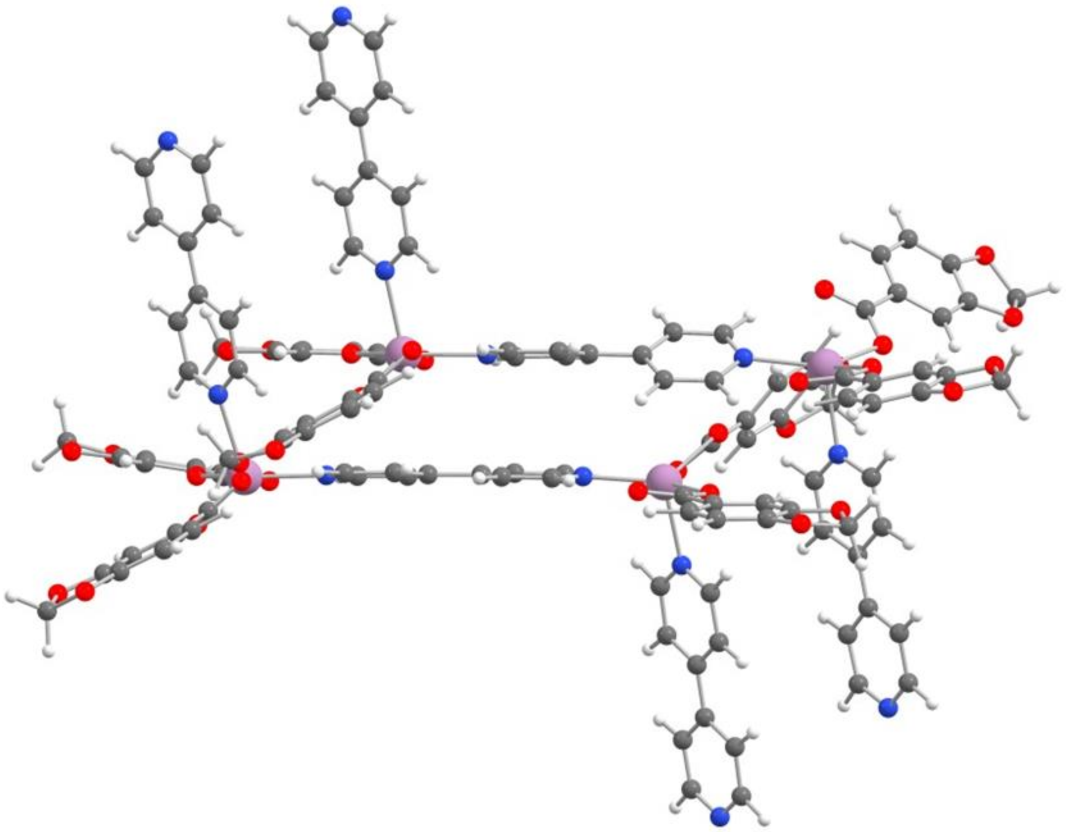

Figure S18. Model used in the energy partitioning scheme. It includes two dimers of vicinal chains in **P1B**.

Solid-state photoluminescence

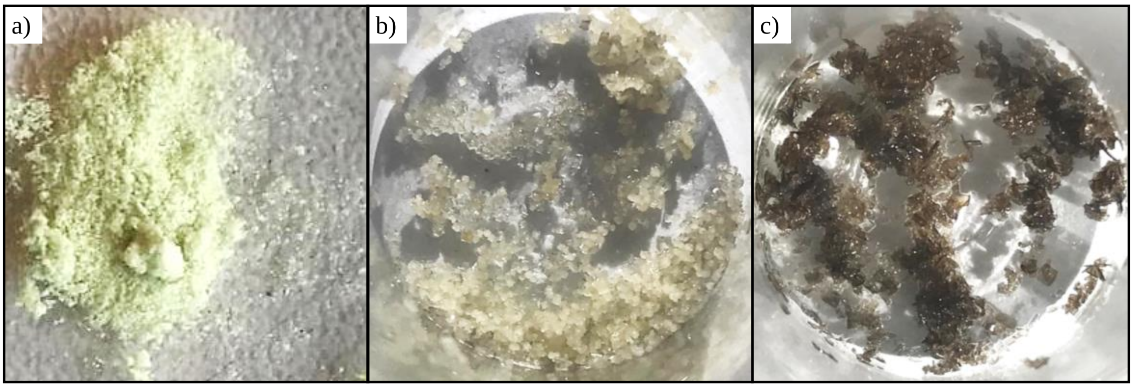

Figure S19. Samples of single crystals of (a) **2**; (b) **P1A** and (c) **P1B** used for the photophysical measurements.

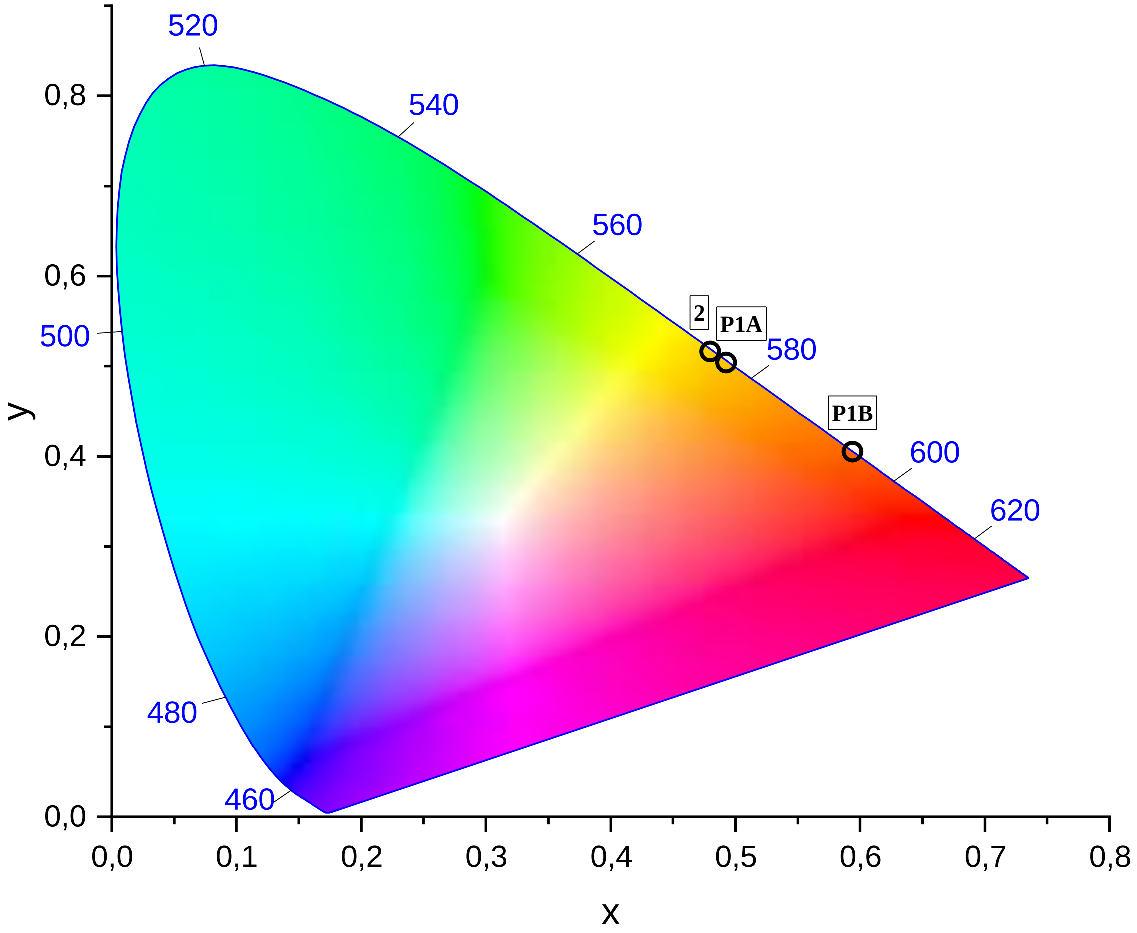

Figure S20. Representation of the emission color of **P1A**, **P1B** and **2** within the CIE 1931 chromaticity diagram.

Table S1. **P1A-P1B** relative energies per [Hg(Pip)$_2$(4,4'-bipy)]·DMF unit formula in kJ mol^{-1} as function of the Grimme's dispersion correction term. A positive value indicates that **P1A** is more stable than **P1B**.

**Method**	**ΔE**
PBE	8.9
PBE-D2	-13.4
[a]PBE-D*	-11.9
PBE-D3	-17.4

[a]PBE-D* stands for a modification of the D2 Grimme's empirical term[1]

Table S2. Unit cell measurements of **P1A** and **P1B** crystals

P1A (structure elucidation)<br>a = 6.0027(7) α = 71.181(4)<br>b = 13.5859(17) β = 83.249(4)<br>c = 17.769(2) γ = 80.217(4)	P1B (structure elucidation)<br>a = 13.6326(15) α = 105.796(4)<br>b = 20.267(2) β = 105.114(4)<br>c = 21.899(2) γ = 100.923(4)
P1A (unit cell measurement)<br>a = 6.00 α = 71.06<br>b = 13.60 β = 83.51<br>c = 17.80 γ = 80.67	P1B (unit cell measurement)<br>a = 13.60 α = 105.75<br>b = 20.21 β = 105.12<br>c = 21.80 γ = 100.94
P1A (unit cell measurement)<br>a = 6.16 α = 70.98<br>b = 13.47 β = 83.12<br>c = 17.69 γ = 80.10	P1B (unit cell measurement)<br>a = 13.69 α = 105.68<br>b = 20.33 β = 105.12<br>c = 21.98 γ = 100.92
P1A (unit cell measurement)<br>a = 6.00 α = 71.65<br>b = 13.70 β = 83.41<br>c = 17.80 γ = 80.26	P1B (unit cell measurement)<br>a = 13.60 α = 105.77<br>b = 20.31 β = 105.20<br>c = 21.86 γ = 100.90

Table S3. Geometry distortions analysis using *S* parameter calculated with SHAPE[2,3]

Compound	*Geometry*[a]	*S value*
P1A	TPR-6	13.467
	OC-6	23.098
	PPY-6	**5.748**
***P1B** (Hg1)*	TPR-6	13.187
	OC-6	20.261
	PPY-6	**7.550**
***P1B** (Hg2)*	TPR-6	12.787
	OC-6	22.941
	PPY-6	**6.450**
***P1B** (Hg3)*	TPR-6	11.386
	OC-6	20.300
	PPY-6	**7.466**
***P1B** (Hg4)*	TPR-6	12.540
	OC-6	22.255
	PPY-6	**5.863**
2	TPR-6	**8.452**
	OC-6	16.523
	PPY-6	18.028

Closer values have been highlighted in bold. [a]TPR-6 = Trigonal prismatic; OC-6 = Octahedral; PPY-6 = Pentagonal pyramidal.

Table S4. Percentage (%) of Hirshfeld surface implied in each contact for compounds **P1A, P1B** and **2**.

**Sample**		**Inter-chain contacts**				**DMF**	
		C···C	Hg[a]	H···O[b]	O···H[b]	H···O	O···H
**P1A**		4.1	3.4	17.0	10.3	20.4[c]	15.2[c]
**P1B**	Hg1	2.7	3.2	16.6	11.4	18.0[d]	14.3[d]
	Hg2	4.1	3.4	16.5	10.7	17.6[e]	14.2[e]
	Hg3	2.7	3.0	16.4	11.4	18.9[e]	15.0[e]
	Hg4	3.8	3.3	16.0	11.0	16.4[f]	14.3[f]
**2**		14.1	0.0	16.4	11.6	-	-

[a]Contacts between Hg(II) centers and aromatic rings. [b] Contacts belonging from C-H···O interactions. dmf molecules interacting with H atoms from [c]Cg1-Cg1; [d]Cg5-Cg5; [e]Cg6-Cg7; or [f]Cg8-Cg8.

References

(1) Civalleri, B.; Zicovich-Wilson, C. M. .; Valenzano, L.; Ugliengo, P. Analysis of the Compression of Molecular Crystal Structures Using Hirshfeld Surfaces. CrystEngComm 2008, 10, 405–410.

(2) Pinsky, M.; Avnir, D. Continuous Symmetry Measures. 5. The Classical Polyhedra. Inorg. Chem. 1998, 37, 5575–5582.

(3) Llunell, M.; Casanova, D.; Cirera, J.; Bofill, J. M.; Alemany, P.; Alvarez, S.; Pinsky, M.; Avnir, D. SHAPE Version 2.1. Universitat de Barcelona and The Hebrew University of Jerusalem 2013.
